# Longitudinal immune characterization of syngeneic tumor models to enable model selection for immune oncology drug discovery

**DOI:** 10.1186/s40425-019-0794-7

**Published:** 2019-11-28

**Authors:** Molly A. Taylor, Adina M. Hughes, Josephine Walton, Anna M. L. Coenen-Stass, Lukasz Magiera, Lorraine Mooney, Sigourney Bell, Anna D. Staniszewska, Linda C. Sandin, Simon T. Barry, Amanda Watkins, Larissa S. Carnevalli, Elizabeth L. Hardaker

**Affiliations:** 1 Oncology R&D, Research and Early Development, Bioscience, AstraZeneca, Francis Crick Ave, Cambridge, CB2 0SL UK; 2 Present Address: Alderley Park Limited, Preclinical Services, Alderley Park, Macclesfield, SK10 4TG UK

**Keywords:** CT-26, MC38, 4 T1, Syngeneic, Immune checkpoint blockade

## Abstract

**Background:**

The ability to modulate immune-inhibitory pathways using checkpoint blockade antibodies such as αPD-1, αPD-L1, and αCTLA-4 represents a significant breakthrough in cancer therapy in recent years. This has driven interest in identifying small-molecule-immunotherapy combinations to increase the proportion of responses. Murine syngeneic models, which have a functional immune system, represent an essential tool for pre-clinical evaluation of new immunotherapies. However, immune response varies widely between models and the translational relevance of each model is not fully understood, making selection of an appropriate pre-clinical model for drug target validation challenging.

**Methods:**

Using flow cytometry, O-link protein analysis, RT-PCR, and RNAseq we have characterized kinetic changes in immune-cell populations over the course of tumor development in commonly used syngeneic models.

**Results:**

This longitudinal profiling of syngeneic models enables pharmacodynamic time point selection within each model, dependent on the immune population of interest. Additionally, we have characterized the changes in immune populations in each of these models after treatment with the combination of α-PD-L1 and α-CTLA-4 antibodies, enabling benchmarking to known immune modulating treatments within each model.

**Conclusions:**

Taken together, this dataset will provide a framework for characterization and enable the selection of the optimal models for immunotherapy combinations and generate potential biomarkers for clinical evaluation in identifying responders and non-responders to immunotherapy combinations.

## Background

The traditional drug development pipeline has relied on testing tumor growth inhibition of human tumor cells in vitro, then testing these molecules in vivo in immunodeficient mice bearing xenografted human tumors [1]. However, this strategy ignores the importance of cross talk between the tumor and other cell types present in the tumor microenvironment (TME), including those of the immune system, that can dramatically impact response to therapy. The ability to modulate immune-inhibitory pathways represents a significant breakthrough in cancer therapy in recent years. Checkpoint blockade antibodies targeting programmed cell-death protein 1 (PD-1), programmed death-ligand 1 (PD-L1), and cytotoxic T-lymphocyte antigen 4 (CTLA-4) have shown great promise in the clinic, causing complete tumor regression and durable responses in a segment of patients [2, 3]. Blockade of the PD-L1/PD1 axis prevents inhibition of T-cell function, while blockade of CTLA-4 induces expansion of tumor reactive T-cells [4, 5] and there is strong interest in identifying small-molecule-immunotherapy combinations to increase the proportion of responses to checkpoint blockade. Identifying the right combinations as well as patients who will respond will rely on building a better understanding of the dynamic interplay between the tumor and the immune system which requires models with a functionally intact immune system. Identification and selection of appropriate in vivo models of immune response requires a better understanding of the dynamic interplay between the tumor and immune system across different models. Syngeneic models represent some of the most established models to investigate immune hypotheses. While several studies have characterized immune populations at single timepoints in syngeneic models, we have sought to characterize kinetic changes in immune populations that occur over time in some of the most commonly used models to better understand the underlying differences in response to immunotherapies.

## Methods

### In vivo studies

All animal studies were performed according to UK Home Office and IACUC guidelines. Cell lines CT-26, 4 T1 and MC38 were purchased from ATCC. CT-26 (5 × 10^5^ cells/mouse) or MC38 (1 × 10^7^ cells/mouse) tumor cells were implanted subcutaneously (s.c.) in the left flank of female Balb/c and C57Bl/6 mice, respectively. 4 T1 (1 × 10^5^ cells/mouse) tumor cells were implanted orthotopically in mammary fat pad 8 of female Balb/c mice. For time course (untreated) studies mice were randomized by body weight on the day of tumor implant, the tumors were collected on day 3 (CT-26 and MC38), day 7 (CT-26, MC38, and 4 T1), day 10 (MC38), day 14 (CT-26 and 4 T1) and day 18 (4 T1). For treated CT-26 studies 5 × 10^5^ cells/mouse were implanted, and mice were randomized by body weight 2 days post implant. For treated MC38 studies 1 × 10^7^ cells/mouse were implanted, and mice were randomized by cage on the day of implant. Mice were injected intraperitoneally with 10 mg/kg of co-formulated α-PD-L1 (mouse IgG1, clone D265A; AstraZeneca) and α-CTLA-4 (mouse IgG1, clone 9D9; AstraZeneca) or the respective isotype controls (αNIP; AstraZeneca) on day 3, 7 and 10 (CT-26) or day 1, 4 and 8 (MC38) post-implant.

### Flow cytometry

At end of study tumor tissues were chopped then transferred into the gentleMACS C Tube containing RPMI. Cells were liberated from tumors for downstream application using a mouse tumor dissociation kit (Miltenyi Biotec) and octodissociator (Miltenyi Biotec) according to manufacturer’s instructions. Cells were stained with a viability marker (Live/Dead Blue, ThermoFisher) according to manufacturer’s instructions and blocked in anti-CD16/CD32 antibody (ThermoFisher). Cells were stained with fluorescence-conjugated antibodies (Additional file 1: Table S1) in flow cytometry staining buffer with Brilliant Stain Buffer (BD Biosciences). Intracellular staining was performed using the FoxP3/transcription factor staining buffer set (ThermoFisher). For extracellular-only panels, cells were fixed in fixation buffer (BD) for 15 min prior to reading. Cells were analyzed on a BD fortessa flow cytometer and analyzed using FlowJo software (V.10, Treestar) or Cytobank. Gating strategies are shown in Additional file 2: Table S2.

### Gene expression analysis and GSVA scoring

Frozen tumors were homogenized using liquid nitrogen and a mortar and pestle to create a powder and 10 mg of tissue was used for RNA isolation by carrying out a Qiazol extraction followed by RNA extraction using the RNeasy Plus Mini Kit with a DNase digestion using the RNase-free DNase Kit (Qiagen) on the Qiacube HT (Qiagen) according to manufacturer’s instructions. RNA concentration was measured using the NanoDrop ND8000 (NanoDrop). Reverse transcription was performed using 50 ng of RNA with a Reverse Transcription kit and cDNA was then pre-amplified (14 cycles) using a pool of TaqMan primers (listed in Additional file 3: Table S3), following the manufacturer’s instructions (Life Technologies). Sample and assay preparation of the 96.96 Fluidigm Dynamic arrays were carried out according to the manufacturer’s instructions. Data were collected and analyzed using Fluidigm Real-Time PCR Analysis 2.1.1 software. dCt was calculated by taking Ct – Average Ct housekeeping genes. An average dCt for all vehicle controls was calculated and (dCt – dCt (average. vehicle)) was used to calculate negative ddCt. 2^negativeddCt was used to calculate Fold Change. *P* values were calculated by performing a student’s t-test on the negative ddCt values in JMP Software and *p* < 0.05 was considered significant. Data were plotted using Spotfire 6.5.3 software or GraphPad Prism (V7). Gene set variation analysis (GSVA) scoring [6] was performed using an in house R script using genes defined in Rooney et al. [7].

### RNAseq

For RNA sequencing, total RNA was extracted using the RNeasy 96 Qiacube HT Kit (Qiagen), quality validated using nanodrop and Quantit RNA Assay Kit (Thermo Fisher), and submitted for TrueSeq Stranded mRNA library preparation, following the manufacturer’s instructions (Illumina). Resulting libraries were sequenced on the HiSeq4000 System, generating on average ~24Million mapped reads. The python toolkit bcbio 1.0.8 (https://github.com/bcbio/bcbio-nextgen) was used to quality control and analyze the sequencing data. In brief, the sequencing reads were aligned using hisat2 2.1.0 [8] for quality control purposes and a QC report was generated using multiqc [9]. Quantification of expression of the transcripts was performed directly against the mouse mm10 Ensembl transcriptome using Salmon 0.9.1 [10] without alignment, or adapter trimming. The R package tximport was used to create a gene by sample count table. Subsequently, the DESeq2 R package (version 1.16.1) was used to normalize for library size and perform differential expression analysis [11].

Genes with an average count of less than 1 per sample were removed. Pathway analysis was performed with IPA QIAGEN Inc., (https://www.qiagenbioinformatics.com/products/ingenuity-pathway-analysis) [12] utilizing fold changes and FDR corrected *p*-values obtained by DESeq2. A customized support vector regression (SVR) model was developed in-house based on the CIBERSORT algorithm to achieve immune cell deconvolution [13]. In brief, this machine learning approach infers the cell type composition of a given tissue sample by hypothesizing a linear relationship between the mixed gene expression profile in the tissue and the expression profile of isolated immune cells provided as reference. Here, we utilized a signature matrix optimized for mouse leukocyte deconvolution to determine the relative proportions of 25 murine immune cell types in the RNA [14].

### O-link proximity extension assay (PEA)

Tumor proteins were lysed in RIPA buffer and diluted to 1 ng/μl before using the Olink mouse exploratory panel (O-link) according to the manufacturer’s instructions. In brief, pairs of oligonucleotide-labeled antibody probes bind to their targeted protein. The oligonucleotides hybridize in a pair-wise manner when brought in close proximity. The addition of a DNA polymerase leads to proximity-dependent DNA polymerization, generating a unique PCR target sequence, which is subsequently detected using a Fluidigm Biomark microfluidic real-time PCR instrument (Fluidigm). The quantification cycle (Cq) values from a DNA extension control are subtracted from the measured cq value and an interplate correction factor applied to yield a normalized protein expression value (NPX), which is log2-transformed.

### Statistics

Error bars relate to SEM unless indicated in figure legends. Appropriate statistical testing was performed using JMP Software, GraphPad Prism (V7) or an in-house R tool. Statistical significance is indicated as follows: * *p* < 0.05, ** *p* < 0.01, *** *p* < 0.001, **** *p* ≤ 0.0001.

## Results

### Benchmarking response to checkpoint blockade in CT-26, MC38 and 4 T1 syngeneic tumor models

**Fig. 1:**
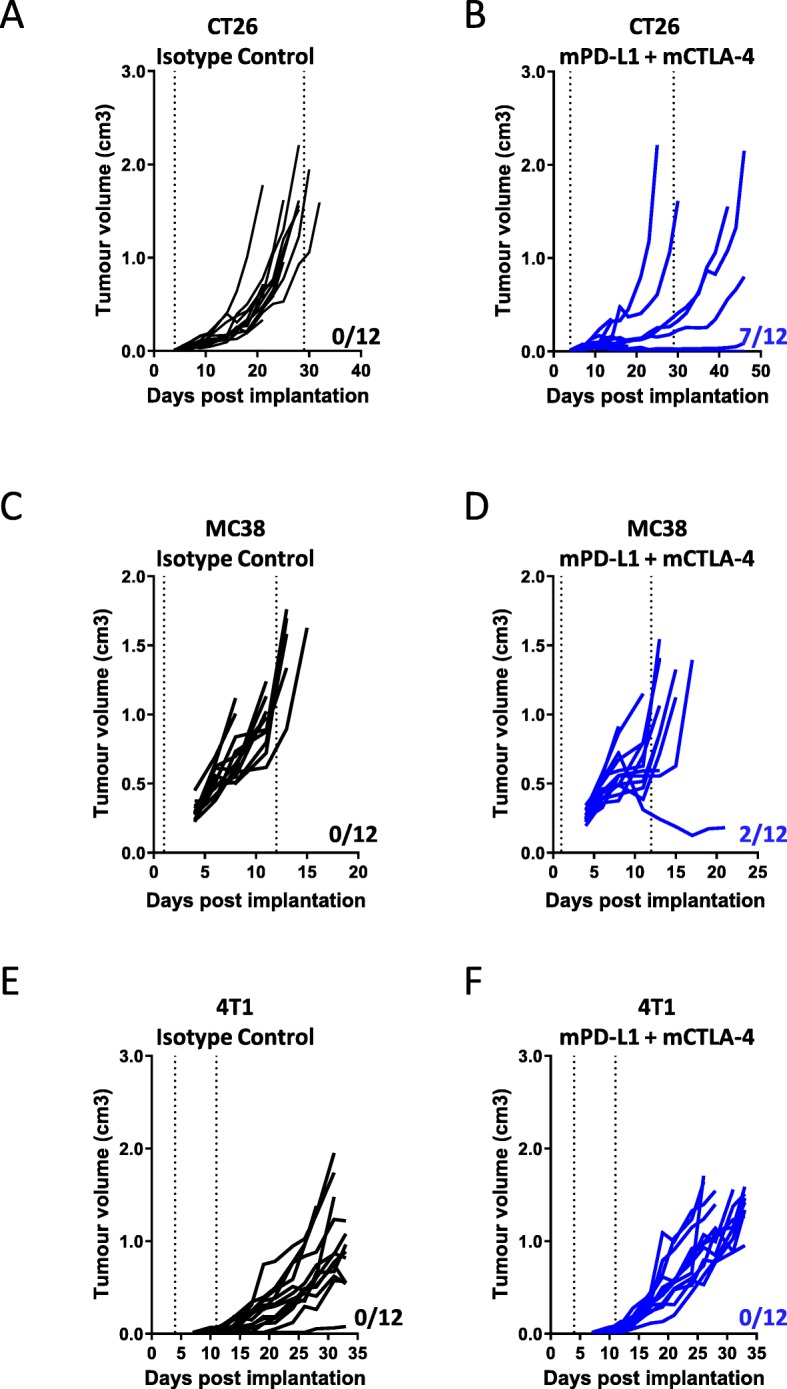
Impact of α-mPD-L1+ α -mCTLA-4 treatment on tumor growth in syngeneic models. Line graphs show tumor volumes from (**a**) Balb/c CT-26 tumor bearing mice treated with isotype control or (**b**) anti-mPD-L1 + anti-mCTLA-4 combination treatment; (**c**) C57Bl/6 MC38 tumor bearing mice treated with Isotype Control or (**d**) anti-mPD-L1 + anti-mCTLA-4 combination treatment; (**e**) Balb/c 4T1 tumor bearing mice treated with isotype control or (**f**) anti-mPD-L1 + anti-mCTLA-4 combination treatment. Vertical dotted lines indicate the period of dosing. *n*=12 per group. Number of responders (those surviving longer than the last vehicle treated) in each model is indicated on each plot

In order to better understand how some of the most commonly utilized syngeneic models respond to checkpoint inhibition, we chose CT-26, MC38, and 4 T1 models for characterization after treatment with a clinically relevant [15] combination of α-mPD-L1 + α-mCTLA-4, which has been shown to induce anti-tumor immune responses in syngeneic models [16]. After tumor implantation, mice were dosed twice a week with a combination of α-PDL-1 + α-CTLA-4 or isotype controls for 2 weeks and tumor growth and survival were measured. In the context of these experiments, the CT-26 model showed the most robust response to checkpoint inhibition (Fig. 1a and b) with 10/12 animals showing reduced tumor growth or stasis leading to an enhanced survival (Additional file 9: Figure S1a). In our hands, the MC38 tumor model showed a more varied response to the same checkpoint inhibition therapy, with delayed tumor growth, but only 1/12 mice showing complete response to therapy (Fig. 1c and d). However, despite only a modest reduction in tumor growth, checkpoint inhibition enhanced survival in this model (Additional file 9: Figure S1b). In contrast to the efficacy observed in CT-26 and MC38 after checkpoint inhibition, the 4 T1 tumor model showed no difference in tumor growth (Fig. 1e and f) and no enhanced survival benefit (Additional file 9: Figure S1c) in response to checkpoint inhibition. All three models expressed PD-L1 in both the myeloid and tumor (CD45-) compartments (Additional file 10: Figure S2). Given this variation in response across these three models observed in our lab and others [17–19], we sought to further characterize the kinetics of immune cell infiltration into the tumor microenvironment over the time course of tumorigenesis in these models as a means to better understand the possible reasons underlying differences in response.

### Longitudinal Immunophenotyping of CT-26 tumors

**Fig. 2:**
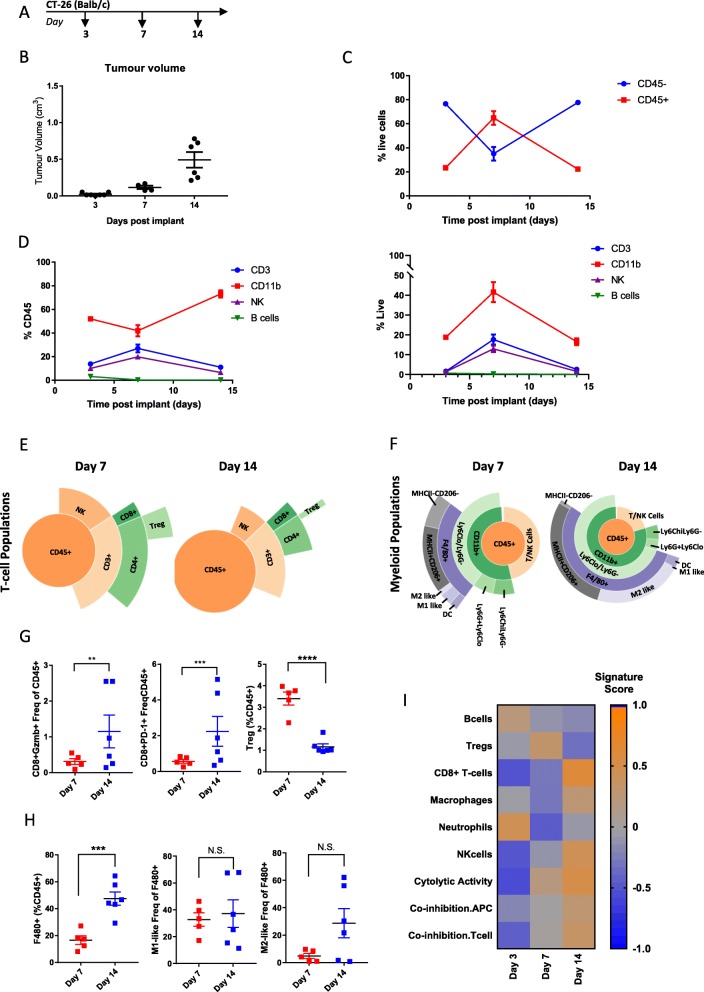
Changes in immune infiltrate over the course of CT-26 tumor development. (**a**) Schematic of sample collection. (**b**) Tumor volumes on indicated day post implant. (**c**) Proportion of CD45- to CD45+ cells measured at each timepoint by flow cytometry (**d**) Proportion of CD3+, CD11b, NK, and B-cells as a percent of CD45+ cells (left) or as a percent of live cells (right) measured by flow cytometry. (**e**) Sunburst blots showing T-cell and NK cell populations as a proportion of CD45+ cells. (**f**) Sunburst plots showing the proportion of myeloid cell populations as a proportion of CD45+ cells. (**g**) Flow cytometry data for individual T-cell populations. (**h**) Flow cytometry data for individual macrophage cell populations. (**i**) Gene expression data generated from a panel of 96 genes was used to calculate a GSVA score [4, 5] indicating enrichment for specific immune cell types at each timepoint. Flow cytometry data is 1 sample from 7 pooled tumors for day 3, 4 tumors from individual animals and 1 sample from 2 pooled tumors on day 7 and 6 individual tumors from day 14. Sunburst plots show data from a pool of *n*=6 samples. For GSVA scores day 3 *n*=4 tumors, day 7 *n*= 6 tumors, and Day 14 *n*=5 tumors. Statistical significance is indicated as NS=not significant, *p<0.05, **p<0.01,***p<0.001, ****p<0.0001. Data for sunburst plots available in Additional file 4: Table S4

CT-26 cells are a colon carcinoma cell line developed by exposing BALB/c mice to N-nitroso-N-methylurethane (NMU), resulting in a grade IV carcinoma that is fast growing and easily implantable [20], making it a workhorse model to study pre-clinical immune mechanisms [21]. This model has previously been characterized as enriched for cytotoxic T-cell and NK cells, using samples taken late in tumor development [17, 19]. In order to explore immune remodeling of the TME during the entire course of tumorigenesis in the CT-26 model, we collected tumors at day 3 when tumors were ~ 25 mm^3^, day 7 when tumors were ~ 100 mm^3^ and day 14 when tumors were ~ 500 mm^3^ (Fig. 2a and b) and performed flow cytometry and gene expression analysis. Examination of total immune infiltrate, measured by infiltration of CD45+ cells, indicated that early, day 3 tumors showed relatively little immune infiltrate (20% CD45+ cells) compared to other tumor/stromal cells (80% CD45-). Interestingly, at day 7 the amount of immune cells (60% CD45+) exceeded the amount of tumor/stromal cells (40% CD45-), which was reversed back to baseline levels by day 14 as the tumors became larger (Fig. 2). Examination of individual immune populations as a percentage of CD45+ cells indicated that the heightened immune infiltrate observed on day 7 was associated with an increased proportion of NK and CD3+ T-cells and a decreased proportion of CD11b + myeloid cells, while B-cells remained low and at a constant level throughout the course of tumorigenesis (Fig. 2d left). Examination of individual immune populations as a percentage of live cells showed similar kinetics, with the exception of myeloid cells which made up a larger proportion of live cells at day 7 (Fig. 2d right). Specifically, we observed that NK cells, CD8+ T-cells, and Tregs reached peak levels at day 7 and had decreased by day 14 (Fig. 2e and Additional file 4: Table S4). In addition to the overall change in T-cell populations, the number of CD8+ T-cells expressing Granzyme B (GzmB+) and PD-1 increased over the course of tumor growth, while the number of Tregs, associated with immune suppression significantly decreased (Fig. 2 g). This is consistent with the strong cytolytic T-cell immune response observed in this tumor model previously [17] and indicates a mechanistic reason why therapies, like checkpoint inhibition, that boost CD8+ T-cell responses work so well in the CT-26 model and suggest that therapies that target Tregs would need to be administered early in this model.

Detailed analysis of the CD11b + myeloid populations revealed that pro-inflammatory M1-like and MHCII-CD206- cells, associated with anti-tumor immune activity remained relatively constant as a proportion of F480+ cells during the course of tumorigenesis. However, there was an increase in total myeloid cells by day 14, which was coupled with a trend towards a higher proportion of M2-like and MHCII+CD206+ macrophages, which are associated with tumor immune suppression and known to promote tumor growth and metastasis (Fig. 2f, h and Additional file 4: Table S4). This is consistent with early anti-tumor immune responses being suppressed as tumors progress and suggests intervention with myeloid-targeting agents might be optimal at or before day 7 in this model.

Using targeted gene expression data, we calculated enrichment scores for immune cell/phenotype signatures (Fig. 2i) [7]. Consistent with the flow cytometry data, this showed enhanced Tregs at day 7 and enhanced macrophages at day 14, with B-cells and neutrophils decreasing through the duration of tumorigenesis (Fig. 2i). In contrast to the flow cytometry data, we observed the peak score for CD8+ T-cells and NK cells at day 14 rather than day 7, which could be due to difference in samples analyzed or differences between gene and protein expression levels. In general, the gene enrichment signatures correlated well with cell population data generated by flow cytometry. Additionally, we observed an increase in gene signatures related to cytolytic activity consistent with the increase in activated T-cells as well as co-inhibition APC and T-cell signatures which is consistent with increasing CD274 (PD-L1) expression and a shift towards immunosuppression that occurs as tumors become larger and more aggressive. Taken together, this data shows that dynamic changes in immune infiltrate occur over the course of CT-26 tumor development.

### Longitudinal Immunophenotyping of MC38 tumors

**Fig. 3:**
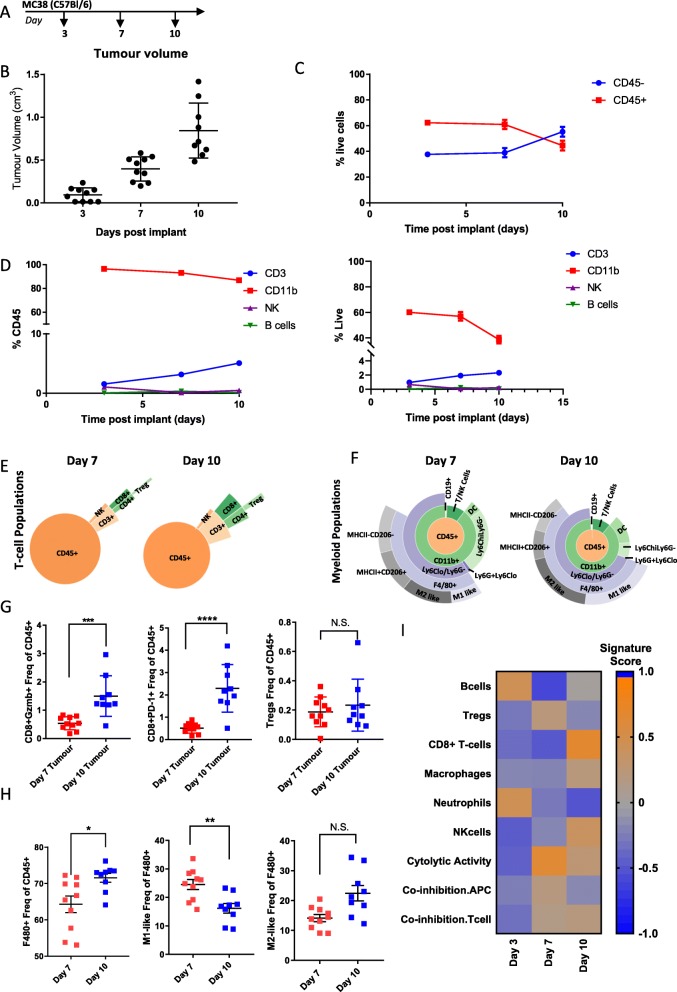
Changes in immune infiltrate over the course of MC38 tumor development. (**a**) Schematic of sample collection. (**b**) Tumor volumes on indicated day post implant. (**c**) Proportion of CD45- to CD45+ cells measured at each timepoint by flow cytometry (**d**) Proportion of CD3+, CD11b, NK, and B-cells as a percent of CD45+ cells (left) or as a percent of live cells (right) measured by flow cytometry. (**e**) Sunburst blots showing T-cell and NK cell populations as a proportion of CD45+ cells. (**f**) Sunburst plots showing the proportion of myeloid cell populations as a proportion of CD45+ cells. (**g**) Flow cytometry data for individual T-cell populations. (**h**) Flow cytometry data for individual macrophage populations (**i**) Gene expression data generated from a panel of 96 genes was used to calculate a GSVA score [4, 5] indicating enrichment for specific immune cell types at each timepoint. Flow cytometry data is 1 sample from 10 pooled tumors for day 3, 10 individual tumors for day 7 and 10 individual tumors for day 14. Sunburst plots show data from a pool of all samples or a representative sample. For GSVA scores *n*=10 for all groups. Statistical significance is indicated as *p<0.05, **p<0.01,***p<0.001, ****p<0.0001. Data for sunburst plots available in Additional file 5: Table S5

MC38 cells are a colon adenocarcinoma cell line derived from C57Bl/6 mice [22]. Similar to our characterization of the CT-26 model, we examined changes in immune infiltrate in the TME throughout the course of tumor development by collecting tumors at day 3 when the tumors were ~ 100 mm^3^, day 7 when the tumors were ~ 400 mm^3^ and day 10 when the tumors were ~ 850 mm^3^ (Fig. 3a and b) and performed detailed flow cytometry and gene expression analysis. Unlike the CT-26 model, where we saw dynamic changes in immune infiltrate throughout the course of tumor development, CD45+ cells remained relatively constant between day 3 and day 7 until shifting to roughly equal proportions of CD45- to CD45+ cells at day 10 when tumors were largest (Fig. 3c). Similarly, the levels of CD11b + myeloid cells and CD3+ T-cells remained fairly constant over the course of tumor growth, with only a slight decrease in myeloid cells and a slight increase in T-cells as a proportion of CD45+ cells at day 10. The number of NK and B-cells as a proportion of CD45+ cells remained low over the entire course of the experiment (Fig. 3d left). Examination of individual populations as a percentage of live cells showed similar trends, with CD11b + cells decreasing and T-cells increasing at Day 10 (Fig. 3d right). A more detailed examination of T-cell populations revealed that unlike CT-26 where we observed expansion of Tregs, NK cells, and CD8+ T-cells at day 7, T-cell and NK populations in MC38 remained stable over the course of tumor development (Fig. 3e, Additional file 5: Table S5). Although there was an overall expansion of myeloid cell types over the course of tumor development, similar to observations in CT-26, this was not associated with a shift from M1-like to M2-like macrophage enrichment (Fig. 3f, Additional file 5: Table S5). Similar to the CT-26 model, MC38 tumors showed a significant increase in GzmB+ and PD-1+ CD8+ T-cells indicative of a cytolytic response to the tumor. However, unlike CT-26, immunosuppressive Tregs did not decrease (Fig. 3 g). The overall increase in F480+ macrophages that occurred during tumor growth was associated with a decrease in the M1-like pro-inflammatory macrophages associated with anti-tumor activity in this model (Fig. 3h). In line with the flow cytometry data, analysis of gene expression signatures indicated an increase in CD8+ T-cells, NK cells and cytolytic activity as well as a modest increase in macrophages over the time course of tumor development (Fig. 3i). Consequently, this data indicates that the MC38 model is characterized by an expansion of T-cell populations and macrophage populations during tumor development. However, the overall changes in immune infiltrate are not as dynamic as those observed in CT-26 (Fig. 2). This suggests that combining checkpoint inhibition with myeloid or Treg targeting agents might enhance efficacy in this model and that perhaps treatment timepoints would be less critical in this model due to the lack of dynamic changes over time.

### Longitudinal Immunophenotyping of 4 T1 tumors

**Fig. 4:**
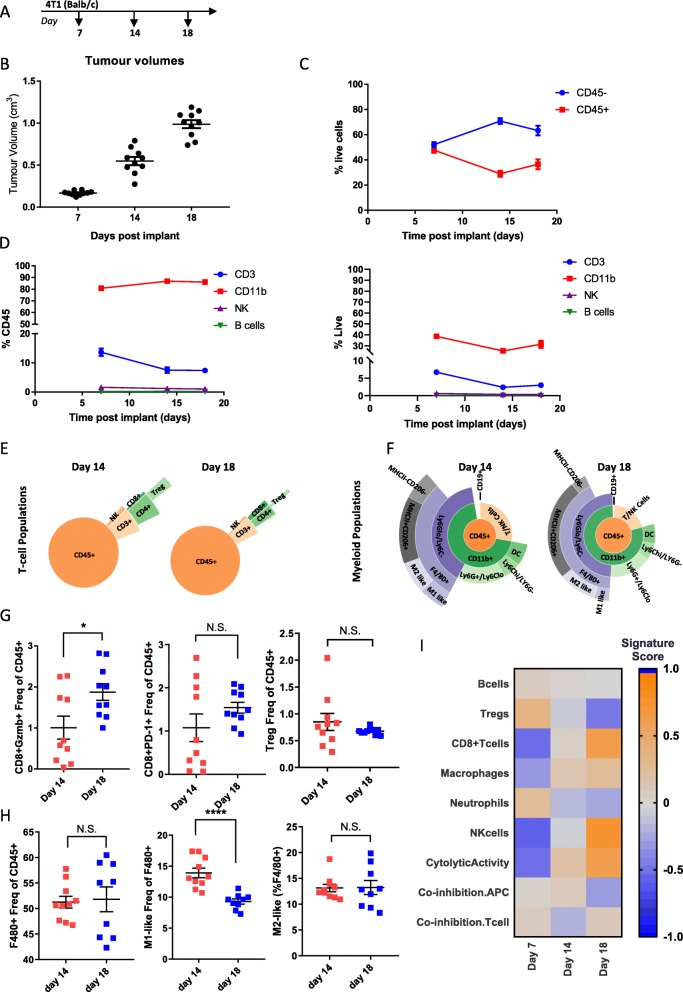
Changes in immune infiltrate over the course of 4T1 tumor development. (**a**) Schematic of sample collection. (**b**) Tumor volumes on indicated day post implant. (**c**) Proportion of CD45- to CD45+ cells measured at each timepoint by flow cytometry (**d**) Proportion of CD3+, CD11b, NK, and B-cells as a percent of CD45+ cells (left) or as a percent of live cells (right) measured by flow cytometry. (**e**) Sunburst plots showing T-cell and NK cell populations as a proportion of CD45+ cells. (**f**) Sunburst plots showing the proportion of myeloid cell populations as a proportion of CD45+ cells. (**g**) Flow cytometry data for individual CD8+ T-cell populations (**h**) Flow cytometry data for individual macrophage populations. (**i**) Gene expression data generated from a panel of 96 genes was used to calculate a GSVA score [4, 5] indicating enrichment for specific immune cell types at each timepoint. Flow cytometry data is *n*=10 for all timepoints. Sunburst plots show data from a pool of samples or a representative sample. For GSVA scores *n*=10 for day 7 and day 14 and *n*=9 for day 18. Statistical significance is indicated as *p<0.05, **p<0.01,***p<0.001, ****p<0.0001. Data for sunburst plots available in Additional file 6: Table S6

4 T1 cells are a highly-metastatic triple negative breast cancer cell line derived from a BALB/c spontaneous mammary carcinoma [23]. Previous studies have shown this model to be highly myeloid enriched and refractory to immune-checkpoint blockade [17, 24], however a detailed examination of populations over time has not been examined. In order to characterize immune populations over the course of tumor development in this model, we collected tumors implanted orthotopically in the mammary fat pad at three timepoints, day 7 when tumors were ~ 170 mm^3^, day 14 when tumors were ~ 550 mm^3^, and day 18 when tumors were ~ 1000 mm^3^ (Fig. 4a and b). In comparison to CT-26 and MC38 models, 4 T1 tumors showed relatively little immune infiltrate, with CD45- cells remaining higher than CD45+ cells throughout the course of tumor growth. Similar to MC38, there were no dynamic changes in the amount of CD45+ cells and they remained relatively constant throughout the course of tumor growth (Fig. 4c). Consistent with 4 T1 tumors being a myeloid enriched model, CD11b + cells comprised the largest proportion of CD45+ immune cells and increased over the course of tumor development, while CD3+ cells decreased, and NK and B-cells remained low over the duration (Fig. 4d left). Examination of immune populations as a proportion of live cells showed a similar pattern to analysis as a proportion of CD45+ cells in this model (Fig. 4d right). A more detailed investigation of individual immune populations indicates that similar to MC38 and unlike CT-26, T-cell populations remain relatively constant over the time course of 4 T1 tumor development, with Tregs slightly decreasing and CD8+ T-cells slightly increasing as tumors progress (Fig. 4e and Additional file 6: Table S6). Detailed investigation of myeloid cell populations indicated that macrophage populations increase over tumor development, consistent with 4 T1 tumors being myeloid enriched (Fig. 4f and Additional file 6: Table S6). Additionally, although there was a small increase in Gzmb+ CD8+ T-cells, there was no increase in PD-1+ CD8+ T-cells and no decrease in Treg cells, indicating much less of a T-cell-mediated immune response to the tumor than that observed in the other two models (Fig. 4 g). Given the high level of F480+ cells in this model it is not surprising that there was no additional increase as tumors progressed (Fig. 4 h). However, interestingly, as tumors progressed there was a significant decrease in the M1-like macrophage population (Fig. 4h), shifting the balance towards the M2-like macrophage population, suggesting that this model may be primed to respond to myeloid targeted therapies. Consistent with the flow cytometry data, gene expression analysis indicated that macrophage populations expanded as 4 T1 tumors progressed. Similar to CT-26 and MC38 CD8+ T-cells, NK cells and cytolytic activity also increased (Fig. 4i). Taken together this data supports evidence that 4 T1 tumors represent a myeloid enriched tumor model which may explain why, despite the expansion in CD8+ T-cells, this model does not respond to checkpoint inhibition.

### Checkpoint inhibition results in changes in immune infiltrate in CT-26

**Fig. 5:**
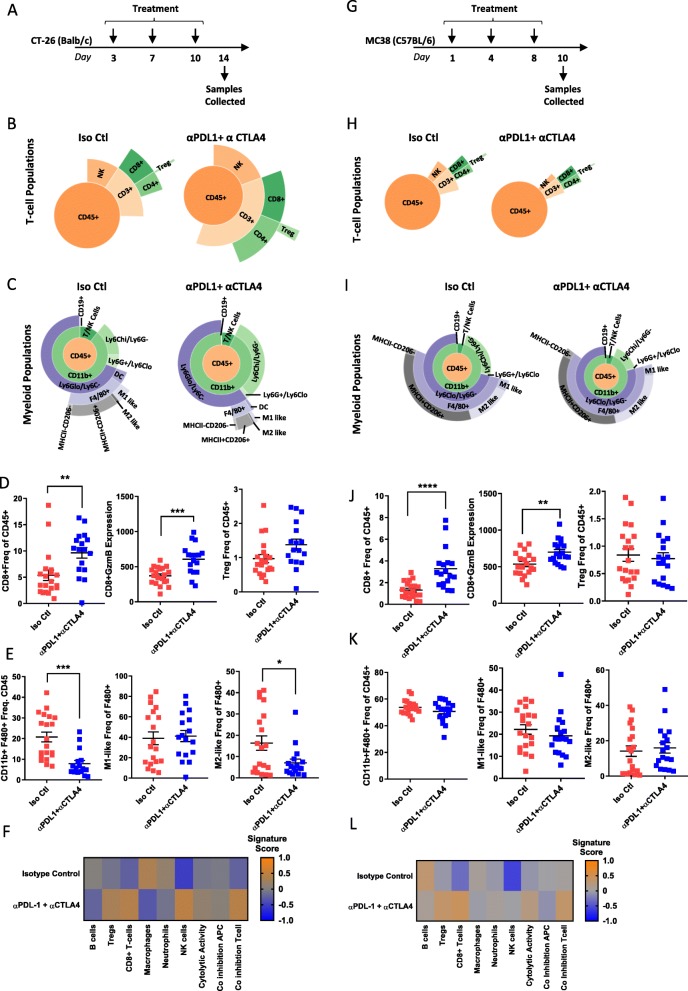
Checkpoint inhibition results in dynamic changes in immune infiltrate in CT-26. (**a**) Schematic of treatment and sample collection in CT-26 model. (**b**) Representative sunburst plot showing T-cell population changes after α-mPD-L1+ α-mCTLA-4 treatment in CT-26 tumors. (**c**) Representative sunburst plot showing changes in myeloid populations after α-mPD-L1+ α-CTLA-4 treatment in CT-26 tumors. (**d**) Flow cytometry data for individual T-cell populations from isotype control treated (*n*=20) or α-mPD-L1+ α-CTLA-4 treated (*n*=17) tumors. (**e**) Flow cytometry data for individual macrophage populations from isotype control treated (*n*=20) or α-mPD-L1+ α-CTLA-4 treated (*n*=17) tumors. (**f**) Gene expression data generated from a panel of 96 genes was used to calculate a GSVA score [4, 5] indicating enrichment for specific immune cell types after treatment with isotype control (*n*=10) or α-mPD-L1+ α-CTLA-4 (*n*=9) in CT-26 tumors. (**g**) Schematic of Treatment and sample collection in MC38 model. (**h**) Representative sunburst plots showing T-cell population changes after α-mPD-L1+ α-mCTLA-4 treatment in MC38 tumors. (**i**) Representative sunburst plots showing changes in myeloid populations after α-mPD-L1+ α-mCTLA-4 treatment in MC38 tumors. (**j**) Flow cytometry data for individual T-cell populations from isotype control treated (*n*=20) or α-mPD-L1+ α-CTLA-4 treated (*n*=18) tumors. (**k**) Flow cytometry data for individual macrophage populations from isotype control treated (*n*=20) or α-mPD-L1+ α-CTLA-4 treated (*n*=18) tumors. (**l**) Gene expression data generated from a panel of 96 genes was used to calculate a GSVA score [4, 5] indicating enrichment for specific immune cell types after isotype control (*n*=6) or α-mPD-L1+ α-CTLA-4 (*n*=9) treatment in MC38 tumors. Data for sunburst plots available in Additional files 7, 8: Tables S7 and S8

As CT-26 and MC38 models showed response to checkpoint inhibition, while 4 T1 did not (Fig. 1), we chose to perform a more detailed immune phenotype characterization after checkpoint therapy in the two responsive models. CT-26 tumors were implanted in Balb/c mice and dosed twice-weekly with α-mPD-L1+ α-mCTLA-4 before collecting tumors on day 14 after treatment (Fig. 5a). Flow cytometry analysis indicated that both CD3+ T-cell and NK cell populations expanded in response to therapy (Fig. 5b), with the total CD3+ T-cell population doubling (Additional file 7: Table S7). In particular, CD8+ T-cells responsible for driving an anti-tumor immune response were significantly increased from 5.37 to 9.64% (Fig. 5b and Additional file 7: Table S7). Coupled with this expansion of cytotoxic T-cells there was a dramatic reduction in all F480+ macrophage populations (Fig. 5c and Additional file 7: Table S7). Consistent with the expansion of CD8+ T-cells and response to therapy, we observed an increase in CD8 + GzmB expression levels as well as a compensatory upregulation of Tregs (Fig. 5d). Additionally, the decrease in F480+ macrophages was associated with a decrease in M2-like macrophages, tipping the balance towards an M1-like pro-inflammatory anti-tumor macrophage response (Fig. 5e). Gene expression analysis phenocopied flow cytometry analysis and indicated that T-cell and NK cell populations expanded after treatment with α-mPD-L1 + α-mCTLA-4, while total macrophage populations were reduced. Furthermore, co-inhibition and cytolytic activity signatures were enriched after treatment (Fig. 5f) indicating an activated T-cell response [25].

Similar to CT-26, MC38 tumors were implanted in C57Bl/6 mice and dosed twice weekly with α-mPD-L1 + α-mCTLA-4 before collecting tumors on day 10 (Fig. 5 g). In comparison to CT-26, MC38 tumors showed a much more modest increase in CD3+ T-cells (Fig. 5 h Additional file 8: Table S8), but still showed a significant increase in CD8+ T-cells which expanded from 1.31 to 3.28% (Additional file 8: Table S8) in response to treatment. Unlike the CT-26 model, MC38 tumors did not show a dramatic reduction in F480+ macrophages (Fig. 5i and Additional file 8: Table S8). Similar to CT-26, checkpoint inhibition in MC38 led to increased GzmB expression in the expanded CD8+ T-cell population (Fig. 5j). However, unlike CT-26, this model did not exhibit a compensatory upregulation in Tregs (Fig. 5j) and did not show any change in overall macrophage populations or shifts in M1-like versus M2-like macrophage levels (Fig. 5k), suggesting a less robust T-cell response and a more suppressive myeloid microenvironment which may explain the less pronounced response to checkpoint inhibition in this model. Mirroring the flow cytometry data, gene expression analysis indicated that T-cell populations increased, and myeloid populations remained stable. Increases in co-inhibition and cytolytic activity signatures were much less pronounced than those observed in CT-26 (Fig. 5 l). Taken together, this data suggests that expansion of tumor-resident T-cells, in particular CD8+ T-cells, coupled with a decrease in myeloid cells is required for response to checkpoint therapy.

### Time course of response to checkpoint inhibition in CT-26

**Fig. 6:**
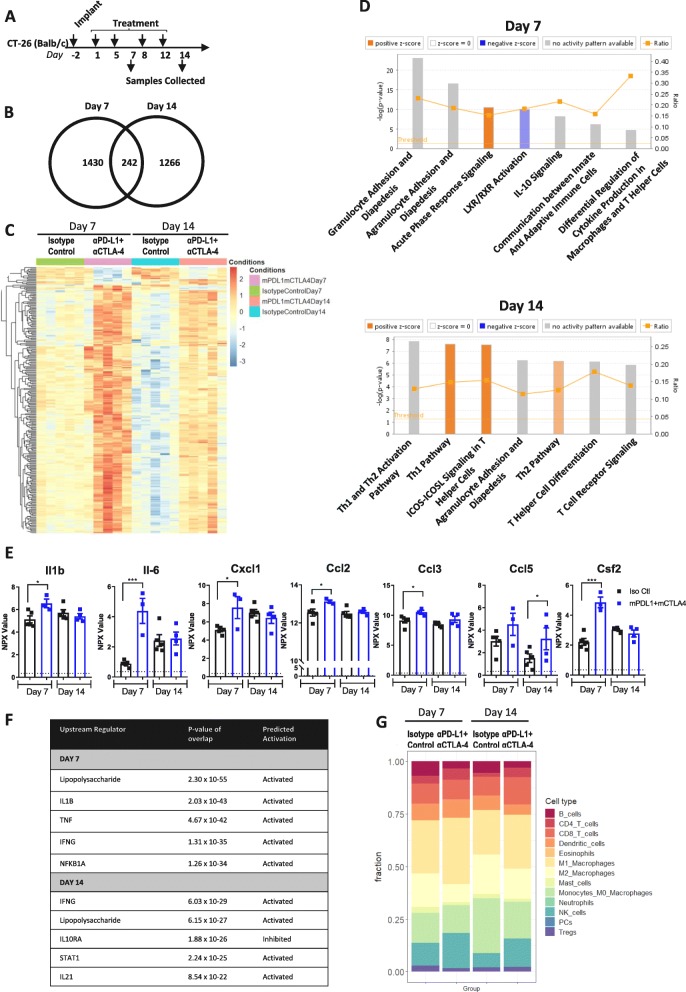
Checkpoint inhibition results in enhanced inflammation and T-cell response in CT-26 syngeneic tumors. (**a**) Schematic of dosing and sample collection. (**b**) Venn diagram indicating the number of genes regulated by checkpoint inhibition at each timepoint. (**c**) Heat map analysis of 242 differentially expressed genes between control and α-mPD-L1 + α-mCTLA-4 treatment at both day 7 and day 14. (**d**) IPA pathway analysis of differentially expressed genes. Z-score indicates a pathway with genes exhibiting overall increase mRNA levels (orange bars) or decreased mRNA levels (blue bars). The ratio (orange line) indicates the ratio of genes from the dataset that map to the same pathway. (**e**) Normalized protein expression (NPX) levels of chemokines measured by O-link PEA assay. (**f**) Upstream regulator pathways from IPA pathway analysis. (**g**) Quantification of immune cellular subtypes based on RNAseq gene signatures between Isotype Control and α-mPD-L1+ α-mCTLA-4 treated samples taken at day 7 and day 14 time points. Statistical significance is indicated as *p<0.05, **p<0.01,***p<0.001, ****p<0.0001, *n*=5 for RNAseq, *n*= 3-5 for PEA

Given the dynamic changes in immune infiltrate observed over the time course of CT-26 tumorigenesis and the strong response to checkpoint inhibition, we sought to more thoroughly characterize the time course of response to checkpoint inhibition in this model. To do so, we treated mice bearing CT-26 tumors twice a week with the combination of α-mPD-L1 + α-mCTLA-4 and collected tumors at day 7 or day 14 post-treatment (Fig. 6a) and performed RNAseq and proximity extension assay (PEA) proteomic analysis (Additional file 11: Figure S3) on the samples. RNAseq analysis indicated that 1672 genes were significantly (p adjusted *p* < 0.05) changed by α-mPD-L1 + α-mCTLA-4 treatment at day 7 and 1508 genes were changed by therapy at day 14 (Fig. 6b). Of these changes, expression of 242 genes were altered by checkpoint inhibition at both timepoints (Fig. 6b & c). Interestingly, samples clustered by day and treatment, not by tumor size (Additional file 12: Figure S4), suggesting that response may rely on the time dependent immune changes we have observed. At day 7, the transcript profile indicated an enrichment for migration of leukocytes in response to inflammation and communication between innate and adaptive immune cells, while at day 14 transcript profiles were enriched for T-helper cell signaling pathways (Fig. 6d). Consistent with this, proteomic analysis indicated that α-mPD-L1 + α-mCTLA-4 treatment resulted in upregulation of chemokines and cytokines associated with inflammation in recruitment of leukocytes at day 7 (IL-6, CXCL1, CCL3, CCL2, Il1β, and CSF2), which returned to control levels by day 14 (Fig. 6e). This supports the idea that α-mPD-L1 + α-mCTLA-4 treatment enhances an early (day 7) inflammatory response, that drives later (day 14) T-cell infiltration and anti-tumor immune response. Similarly, upstream pathway analysis of the transcriptome data indicated that lipopolysaccharide (LPS), IL-1B, TNF, IFNG, and NFKB1A pathways associated with inflammation were activated at day 7 (Fig. 6f). However, by day 14, although LPS and IFNG pathways remained activated, this was coupled with STAT1 and IL21 pathway enrichment indicative of innate and adaptive immune response to inflammation [26]. Examination of lymphocyte subtype fractions using transcript expression indicated that coupled with the enhanced inflammation induced by α-mPD-L1 + α-mCTLA-4 treatment, there was an increase in CD8+ T-cells, NK-cells, and M1-like (pro-inflammatory) macrophages (Fig. 6 g), suggesting that these cell types are responsible for driving the responses observed in the CT-26 model. In this particular study there were not enough animals to dissect differences in responder and non-responder mice, so we performed a larger CT-26 study and examined differences in immune cell content between responders and non-responders. Interestingly, there was no difference in T-cell content between responders and non-responders (Additional file 13: Figure S5a, S5b, S5c). However, frequency of CD11b + and F480+ myeloid cells was significantly higher in non-responders (Additional file 13: Figure S5d and S5e). This supports our observations, and others [27], that expansion of myeloid cells during tumor progression (Fig. 2f , h) may suppress response to checkpoint blockade and that eliminating the suppressive myeloid populations (e.g. macrophages) is important for eliciting anti-tumor response to checkpoint blockade. Taken together, this suggests that while α-mPD-L1 + α-mCTLA-4 treatment drives an expected T-cell activation, it is the myeloid content of the tumor that correlates and possibly determines anti-tumor response. Further studies would be needed to determine the mechanistic role of myeloid populations in response to immunotherapy.

## Discussion

It is becoming increasingly clear that modulation of the immune system represents a successful therapeutic strategy for combating cancer. Given the variability in response to immune-targeted therapies that is observed both pre-clinically and clinically, a greater understanding of pre-clinical models will facilitate development of new immune-modulatory agents and combination strategies. Syngeneic models are some of the most readily available, fast-growing, and high-throughput tumor model systems available to address pre-clinical questions. However, syngeneic models can possess limitations in term of translatability to human tumors. These tumors do not develop spontaneously, often do not contain the microenvironment of the tumor of origin, and progress much more rapidly than spontaneous tumors. Indeed, GEMM (genetically engineered mouse models) often represent more physiologically relevant models of human tumor progression as they contain disease relevant mutations and better recapitulate the multi-step process of tumor progression [28]. Despite their limitations, syngeneic models currently represent the best models for carrying out large scale studies to investigate mechanistic immune changes and drug mode of action. It is known that Balb/c mice preferentially trigger a strong Th2 response, whereas C57Bl/6 mice preferentially trigger a Th1 response to mycobacterial vaccination [29, 30]. However, given that we see a strong anti-tumor response in CT-26 and a weak anti-tumor response in 4 T1, both in Balb/c, we cannot conclude that differences in anti-tumor immune response are due to mouse strain. However, a wider study of multiple models in both backgrounds would be needed to investigate any anti-tumor immune response differences dependent on background strain. Here we have presented an overview of how the tumor-immune microenvironment evolves during the course of tumor development in CT-26, MC38 and 4 T1, three of the most commonly used syngeneic models. Immune changes in CT-26, the model most responsive to checkpoint inhibition, were dynamic through the course of tumor development and T-cell infiltration and cytolytic response was greatly enhanced with the addition of α-mPDL1+ α-mCTLA-4 treatment. Moreover, this model had a reduction in CD11b + myeloid cells, which was associated with response to checkpoint inhibition, suggesting that dynamic changes in multiple cellular compartments may be required to elicit efficacy. In contrast to CT-26, MC38 and 4 T1 models had very little change in immune infiltrate throughout the course of tumor development and were heavily enriched with immunosuppressive cell types. Even in response to treatment with α-mPDL1+ α-mCTLA-4 MC38 tumors were characterized by only a modest increase in T-cells and very small reductions in myeloid cell populations. This suggests that in addition to expansion of cytotoxic T-cell populations, reduction of myeloid cells may be important for robust response to checkpoint inhibition, an observation that is further supported by recent findings that myeloid remodeling is necessary for efficient response to checkpoint inhibitors [27]. Moreover, depleting myeloid cells by targeting growth factor receptor CSF1R or CXCR2 has shown modest efficacy in subcutaneous models. However, agents that reprogram myeloid cells, such as PI3Kγ inhibitors, are more effective in combination with checkpoint inhibition. It is unclear why reversing pro-inflammatory myeloid phenotypes is more effective, but may indicate that myeloid cell function and regulation is highly context dependent, and may be related to the role in the local micro-environment [31, 32]. Our observations suggest that the dynamic changes in immune infiltrate observed in CT-26 tumors may be a driving factor in the positive responses to immunotherapy observed in this model [17, 33].

A better understanding of the tumor microenvironment and how it responds to checkpoint blockade is paramount to designing rational IO combinations to provide better therapeutic margins. Moreover, pharmacodynamic changes do not always match efficacy readouts. In order to choose appropriate timepoints to measure pharmacodynamic readouts for target immune populations a better understanding of the kinetics of these changes is needed. The work presented here shows longitudinal changes in the tumor microenvironment of key preclinical tumor models. This information fills a gap in current understanding of longitudinal immune response and provides a key reference data set for future experiments.

## Conclusions

We provide immune characterization of syngeneic tumors during the time course of tumor development as well as characterization of models that respond to checkpoint therapy which will enable benchmarking of novel immunotherapies to well characterized checkpoint inhibitors and identification of biomarkers of response.

## Supplementary information

**Additional file 1: Table S1.** Antibody panels.

**Additional file 2: Table S2.** Gating strategy.

**Additional file 3: Table S3.** Primer information.

**Additional file 4: Table S4.** CT-26 timecourse flow data.

**Additional file 5: Table S5.** MC38 timecourse flow data.

**Additional file 6: Table S6.** 4T1 timecourse flow data.

**Additional file 7: Table S7.** CT-26 checkpoint treatment flow data.

**Additional file 8: Table S8.** MC38 checkpoint treatment flow data.

**Additional file 9: Figure S1**. Impact of α-mPD-L1+ α -mCTLA-4 treatment on survival in syngeneic models.

**Additional file10: Figure S2.** Expression of PD-L1 on CD11b+ and CD45- cells.

**Additional file 11: Figure S3.** Protein expression changes measured by NPX

**Additional file 12: Figure S4.** Impact of tumor size on gene expression changes in CT-26

**Additional file 13: Figure S5.** Immune cell content in CT-26 responders and non-responders

## Data Availability

The authors declare that data supporting the findings of this study are available within the article and its supplementary information files. RNAseq data is available through ArrayExpress. E-MTAB-7777.

## Abbreviations

CTLA-4: Cytotoxic T-lymphocyte antigen 4
GEMM: Genetically engineered mouse models
GzmB: Granzyme B
LPS: Lipopolysaccharide
NMU: N-nitroso-N-methylurethane
PD-1: Programmed cell-death protein 1
PD-L1: Programmed death-ligand 1
TME: Tumor microenvironment
