# Supplementary material for: Longitudinal immune characterization of syngeneic tumor models to enable model selection for immune oncology drug discovery
Source: J Immunother Cancer. 2019 Nov 28;7:328. doi: 10.1186/s40425-019-0794-7 (PMC6883640; doi:10.1186/s40425-019-0794-7)
Supplement: Supplementary file 1 — Additional file 1: Table S1. Antibody panels. [file 40425_2019_794_MOESM1_ESM.docx]

**Supplementary**

**Supplementary Table 1-Ab panels**

**T-cell panel**

| Antibody | Fluorochrome | Vendor | Cat No. | Clone | Final dilution | location |
| --- | --- | --- | --- | --- | --- | --- |
| live dead | UV | Invitrogen | L23105 | fixable blue | 1:400 | intracellular- unfixed |
| CD45 | BV786 | BD | 564225 | 30-F11 | 1:1000 | extracellular |
| CD3 | BUV395 | BD | 740268 | 17A2 | 1:200 | extracellular |
| CD4 | BV711 | Biolegend | 100557 | RM4-5 | 1:800 | extracellular |
| CD8a | BV650 | Biolegend | 100742 | 53-6-7 | 1:800 | extracellular |
| NKp46 | BV605 | Biolegend | 137619 | 29A14 | 1:100 | extracellular |
| CD25 | PE/Cy7 | Biolegend | 102016 | PC61 | 1:800 | extracellular |
| CD44 | BUV737 | BD | 564392 | 1M7 | 1:200 | extracellular |
| CD62L | PE-CF594 | BD | 562404 | MEL14 | 1:200 | extracellular |
| PD-1 | BV421 | Biolegend | 135218 | 29F.1.A12 | 1:800 | extracellular |
| Foxp3 | APC | Ebioscience | 17-5773-82 | FJK-16S | 1:200 | intracellular |
| GzmB | PE | Thermo | MHGB04 | GB12 | 1:200 | intracellular |
| Ki67 | Alexa488 | Biolegend | 151204 | 11F6 | 1:200 | intracellular |

**Myeloid panel**

| Antibody | Fluorochrome | Vendor | Cat No. | Clone | Final dilution | location |
| --- | --- | --- | --- | --- | --- | --- |
| live/ dead | UV | Invitrogen | L23105 | fixable blue | 1:400 | intracellular- unfixed |
| CD45 | BV786 | BD | 564225 | 30-F11 | 1:1000 | extracellular |
| CD11b | BUV395 | BD | 563553 | M1/70 | 1:200 | extracellular |
| F4/80 | APC | Biolegend | 123116 | BM8 | 1:200 | extracellular |
| CD11c | PE | ebioscience | 12-0114-83 | N418 | 1:200 | extracellular |
| MHCII | AF700 | Biolegend | 107622 | M5/114.15.2 | 1:400 | extracellular |
| Ly6G | APC/Cy7 | Biolegend | 127624 | 1A8 | 1:400 | extracellular |
| Ly6C | PerCP/Cy5.5 | Biolegend | 128012 | HK1.4 | 1:400 | extracellular |
| PDL-1 | BV421 | Biolegend | 124315 | 10F9G2 | 1:800 | extracellular |
| CD64 | PE/Dazzle | Biolegend | 139319 | X54-5/7.1 | 1:400 | extracellular |
| CD206 | PE/Cy7 | Biolegend | 141719 | C068C2 | 1:1000 | extracellular |
| CD19 | BUV737 | BD | 564296 | ID3 | 1:200 | extracellular |

**T and myeloid panel**

| Antibody | Fluorochrome | Vendor | Cat No. | Clone | Final dilution | location |
| --- | --- | --- | --- | --- | --- | --- |
| live/ dead | UV450 /DAPI | Thermo | L-23105 |  | 1:400 | intracellular- unfixed |
| CD45 | BV785/6 | BD | 564225 | 30-F11 | 1:1000 | extracellular |
| CD3 | BUV395 | BD | 740268 | 17A2 | 1:200 | extracellular |
| CD4 | BV711 | Biolegend | 100557 | RM4-5 | 1:800 | extracellular |
| CD8a | BV650 | Biolegend | 100742 | 53-6-7 | 1:800 | extracellular |
| CD25 | PE-Cy7 | Biolegend | 102016 | PC61 | 1:800 | extracellular |
| CD19 | BUV737 | BD | 564296 | ID3 | 1:200 | extracellular |
| NKp46 | BV605 | Biolegend | 137619 | 29A14 | 1:100 | extracellular |
| CD11b | Pe CF594 | BD | 562287 | M1/70 | 1in800 | extracellular |
| F4/80 | BV510 | Biolegend | 123135 | BM8 | 1in200 | extracellular |
| Ly6G | APC-Cy7 | Biolegend | 127624 | 1A8 | 1:400 | extracellular |
| Ly6C | PerCP-Cy55 | Biolegend | 128012 | HK1.4 | 1:400 | extracellular |
| CD11c | PE | ebioscience | 12-0114-83 | N418 | 1:200 | extracellular |
| MHCII | AF700 | Biolegend | 107622 | M5/114.15.2 | 1:400 | extracellular |
| PD-1 | BV421 | Biolegend | 135218 | 29F.1.A12 | 1:800 | extracellular |
