# Supplementary material for: Longitudinal immune characterization of syngeneic tumor models to enable model selection for immune oncology drug discovery
Source: J Immunother Cancer. 2019 Nov 28;7:328. doi: 10.1186/s40425-019-0794-7 (PMC6883640; doi:10.1186/s40425-019-0794-7)
Supplement: Supplementary file 2 — Additional file 2: Table S2. Gating strategy. [file 40425_2019_794_MOESM2_ESM.docx]

**Supplementary Table 2**

| **Flow cytometry population** | **Cell Type** |
| --- | --- |
| Dead / live | Viability |
| CD45+ | hematopoietic cells |
| CD45+NKp46+ | NK cells |
| CD45+ CD3+ | All T-cells |
| CD45+ CD3+ CD4+ | Total CD4+ T-cell |
| CD45+ CD3+ CD4+ CD25+ FoxP3+ | T regulatory |
| CD45+ CD3+ CD4+ CD62L+ CD44- | Naive |
| CD45+ CD3+ CD4+CD62L- CD44+ | Effector/Memory |
| CD45+ CD3+ CD4+ CD62L+CD44+ | Central Memory T-cells |
| CD45+ CD3+ CD8+ | Cytotoxic T-cell |
| CD45+ CD3+ CD8+ CD62L+ CD44- | Naive |
| CD45+ CD3+ CD8+ CD62L- CD44+ | Effector/Memory |
| CD45+ CD3+ CD8+ CD62L+CD44+ | Central Memory T-cells |
| GzmB | Cytotoxic T-cell marker |
| Ki67 | Proliferation marker |
| PD-1 | T-cell Activation/exhaustion marker |
| PD-L1 | Programmed death-ligand 1 |
| CD45+CD19+ | B cells |
| CD45+ CD11b+ | Myeloid |
| CD45+ CD11b+F4/80+ | Macrophages |
| CD45+ CD11b+F4/80+ MHCII-CD206+ | M2 like macrophages |
| CD45+ CD11b+F4/80+MHCII+CD206- | M1 like macrophages |
| CD45+CD11b+Ly6Ghi Ly6Clo | Granulocytes/ gMDSC |
| CD45+CD11b+Ly6G- Ly6Chi | moMDSC |
| CD45+CD11b+CD11c+MHCIIhi | Dendritic cells |
| CD64+ | Myeloid activation marker |
| Other in Myeloid panel | CD11b-CD19- |
| Other in T panel | CD3-NK- |
