# Supplementary material for: Longitudinal immune characterization of syngeneic tumor models to enable model selection for immune oncology drug discovery
Source: J Immunother Cancer. 2019 Nov 28;7:328. doi: 10.1186/s40425-019-0794-7 (PMC6883640; doi:10.1186/s40425-019-0794-7)
Supplement: Supplementary file 3 — Additional file 3: Table S3. Primer information. [file 40425_2019_794_MOESM3_ESM.docx]

**Supplementary Table 3**

| **Gene Symbol** | **Assay ID** |
| --- | --- |
| 18S | Hs99999901_s1 |
| Actb | Mm00607939_s1 |
| Ahr | Mm00478932_m1 |
| Btla | Mm00616981_m1 |
| Foxp3 | Mm00475164_m1 |
| Ctla4 | Mm00486849_m1 |
| Il5 | Mm00439646_m1 |
| Cd160 | Mm00444461_m1 |
| Cd2 | Mm00488928_m1 |
| Cd226 | Mm01301769_m1 |
| Cd244 | Mm00479575_m1 |
| Cd27 | Mm01185212_g1 |
| Cd79b | Mm00434143_m1 |
| Cd28 | Mm01253994_m1 |
| Cd40 | Mm00441891_m1 |
| Cd40lg | Mm00441911_m1 |
| Cd8a | Mm01182108_m1 |
| Cd68 | Mm03047343_m1 |
| Cd70 | Mm00441914_m1 |
| Btla | Mm00616981_m1 |
| B2m | Mm00437762_m1 |
| Clec5a | Mm01131767_m1 |
| Fcgr4 | Mm00519988_m1 |
| Cxcr3 | Mm99999054_s1 |
| Cybb | Mm01287743_m1 |
| Ddx4 | Mm00802445_m1 |
| Gm21975;Evi2b | Mm00524622_m1 |
| Bank1 | Mm01317729_m1 |
| Fuca1 | Mm00502778_m1 |
| Gapdh | Mm99999915_g1 |
| Gpnmb | Mm01328587_m1 |
| Gpr146 | Mm01951835_s1 |
| Gpr15 | Mm03990531_s1 |
| Gzma | Mm01304452_m1 |
| Cd79a | Mm00432423_m1 |
| Havcr2 | Mm00454540_m1 |
| Hprt | Mm03024075_m1 |
| Hs3st2 | Mm03038522_m1 |
| Hsd17b11 | Mm00504410_m1 |
| Blk | Mm00432077_m1 |
| Icosl | Mm00497237_m1 |
| Ifit1 | Mm00515153_m1 |
| Ifit2 | Mm00492606_m1 |
| Ifit3 | Mm01704846_s1 |
| Tap1 | Mm00443188_m1 |
| Il3ra | Mm00434273_m1 |
| Ralgps2 | Mm01286206_m1 |
| Fcrl1 | Mm01218088_m1 |
| Ipo8 | Mm01255165_m1 |
| Irf7 | Mm00516793_g1 |
| Hvcn1 | Mm01199507_m1 |
| Isg20 | Mm00469585_m1 |
| Kdm6b | Mm01332680_m1 |
| Klrc1 | Mm00516111_m1 |
| Cd274 | Mm00452054_m1 |
| Lag3 | Mm00493071_m1 |
| Lair1 | Mm00618113_m1 |
| Lgals9 | Mm00495295_m1 |
| Lgmn | Mm01325350_m1 |
| Prf1 | Mm00812512_m1 |
| Megf9 | Mm00554571_m1 |
| Bach2 | Mm00464379_m1 |
| Ifi205,Mnda | Mm04204353_mH |
| H2-Aa | Mm00439211_m1 |
| Mx2 | Mm00488995_m1 |
| Nlrp12 | Mm01329688_m1 |
| Padi4 | Mm01341658_m1 |
| Pdcd1lg2 | Mm00451734_m1 |
| Phex | Mm00448119_m1 |
| Pld4 | Mm00626861_m1 |
| Ppia | Mm03302254_g1 |
| H2-Ab1 | Mm00439216_m1 |
| Ptcra | Mm00478363_m1 |
| Ptprc | Mm01293577_m1 |
| Pvrl3 | Mm01342993_m1 |
| Rsad2 | Mm00491265_m1 |
| Sell | Mm00441291_m1 |
| Selp | Mm01295931_m1 |
| Slamf1 | Mm00443316_m1 |
| Tigit | Mm03807522_m1 |
| Tm4sf19 | Mm01344503_m1 |
| Tnfrsf18 | Mm00437136_m1 |
| Tnfrsf25 | Mm00459629_g1 |
| Tnfrsf4 | Mm00442039_m1 |
| Tnfrsf8 | Mm00437140_m1 |
| Tnfrsf9 | Mm00441899_m1 |
| Hprt | Mm01545399_m1 |
| Tnfsf14 | Mm00444567_m1 |
| Tnfsf15 | Mm00770031_m1 |
| Tnfsf18 | Mm00839222_m1 |
| Tnfsf4 | Mm00437214_m1 |
| Tnfsf8 | Mm00437153_m1 |
| Tnfsf9 | Mm00437155_m1 |
| Trank1 | Mm01245649_m1 |
| Vnn3 | Mm00496418_m1 |
| Ywhaz | Mm01158416_g1 |
