# Supplementary material for: Longitudinal immune characterization of syngeneic tumor models to enable model selection for immune oncology drug discovery
Source: J Immunother Cancer. 2019 Nov 28;7:328. doi: 10.1186/s40425-019-0794-7 (PMC6883640; doi:10.1186/s40425-019-0794-7)
Supplement: Supplementary file 4 — Additional file 4: Table S4. CT-26 timecourse flow data. [file 40425_2019_794_MOESM4_ESM.docx]

**Supplementary Table 4**

| **CT-26** | **Day 7 (n=6)** | | **Day 14 (n=6)** | |
| --- | --- | --- | --- | --- |
| **T-cell Panel** | **mean** | **SE** | **mean** | **SE** |
| Live (%singlets) | 88.560 | 3.729 | 98.733 | 0.194 |
| CD45+ (%live) | 64.880 | 5.692 | 22.317 | 1.465 |
| CD3+ (%CD45+) | 28.300 | 3.443 | 6.743 | 0.727 |
| CD4+ (%CD45+) | 17.640 | 1.977 | 4.048 | 0.306 |
| Treg (%CD45+) | 3.406 | 0.299 | 1.165 | 0.106 |
| CD8+ (%CD45+) | 2.944 | 0.416 | 1.168 | 0.272 |
| NK (%CD45+) | 15.880 | 1.237 | 6.728 | 1.073 |
| **Myeloid Panel** | **mean** | **SE** | **mean** | **SE** |
| Live (%singlets) | 89.440 | 2.944 | 98.250 | 0.407 |
| CD45+(%live) | 66.700 | 4.182 | 20.267 | 2.341 |
| CD11b+ (%CD45+) | 61.600 | 4.492 | 86.883 | 2.047 |
| M-MDSC | 14.260 | 1.466 | 11.430 | 1.908 |
| Ly6G-Ly6Clo | 39.400 | 2.992 | 70.300 | 4.785 |
| Macrophages (%CD45+) | 16.638 | 3.204 | 47.533 | 4.985 |
| M1 like (%CD45+) | 0.602 | 0.185 | 16.047 | 6.741 |
| M2 like (%CD45+) | 5.858 | 1.887 | 15.543 | 3.392 |
| MHCII+CD206+ (%CD45) | 3.882 | 0.860 | 11.783 | 1.469 |
| MHCII-CD206- (%CD45) | 6.310 | 1.046 | 4.145 | 1.205 |
| G-MDSC (%CD45+) | 4.906 | 0.951 | 4.383 | 0.831 |
| DC (%CD45+) | 1.718 | 0.473 | 3.757 | 0.336 |
| B cells (%CD45+) | 0.484 | 0.108 | 0.348 | 0.027 |
