# Supplementary material for: Longitudinal immune characterization of syngeneic tumor models to enable model selection for immune oncology drug discovery
Source: J Immunother Cancer. 2019 Nov 28;7:328. doi: 10.1186/s40425-019-0794-7 (PMC6883640; doi:10.1186/s40425-019-0794-7)
Supplement: Supplementary file 5 — Additional file 5: Table S5. MC38 timecourse flow data. [file 40425_2019_794_MOESM5_ESM.docx]

**Supplementary Table 5**

| **MC38** | **Day 7 (n=10)** |  | **Day 10 (n=9)** |  |
| --- | --- | --- | --- | --- |
| **T-cell Panel** | **mean** | **SE** | **mean** | **SE** |
| Live (%singlets) | 84.690 | 1.921 | 90.756 | 1.243 |
| CD45+ (%live) | 60.980 | 3.652 | 49.278 | 3.312 |
| CD3+ (%CD45+) | 3.551 | 0.196 | 5.194 | 0.493 |
| CD4+ (%CD45+) | 1.002 | 0.073 | 2.072 | 0.178 |
| Treg (%CD45+) | 0.188 | 0.032 | 0.234 | 0.059 |
| CD8+ (%CD45+) | 1.195 | 0.093 | 2.128 | 0.316 |
| NK (%CD45+) | 3.639 | 0.408 | 2.159 | 0.244 |
| **Myeloid Panel** | **mean** | **SE** | **mean** | **SE** |
| Live (%singlets) | 89.03 | 6.41 | 92.79 | 2.09 |
| CD45+(%live) | 56.54 | 16.76 | 42.73 | 6.55 |
| CD11b+ (%CD45+) | 90.00 | 1.19 | 84.75 | 3.08 |
| M-MDSC (%CD45) | 21.38 | 6.05 | 9.96 | 2.46 |
| Ly6G-Ly6Clo (%CD45) | 65.36 | 6.20 | 73.44 | 3.55 |
| Macrophages (%CD45+) | 51.61 | 7.58 | 63.98 | 4.25 |
| M1 like (%CD45+) | 11.94 | 3.29 | 11.86 | 4.57 |
| M2 like (%CD45+) | 11.06 | 3.42 | 17.40 | 7.16 |
| MHCII+CD206+ (%CD45) | 11.17 | 2.46 | 20.83 | 4.85 |
| MHCII-CD206- (%CD45) | 17.44 | 3.98 | 13.89 | 3.11 |
| G-MDSC (%CD45+) | 1.28 | 0.54 | 0.37 | 0.12 |
| DC (%CD45+) | 4.60 | 1.17 | 10.32 | 3.12 |
| B cells (%CD45+) | 0.45 | 0.20 | 0.40 | 0.10 |
