# Supplementary material for: Longitudinal immune characterization of syngeneic tumor models to enable model selection for immune oncology drug discovery
Source: J Immunother Cancer. 2019 Nov 28;7:328. doi: 10.1186/s40425-019-0794-7 (PMC6883640; doi:10.1186/s40425-019-0794-7)
Supplement: Supplementary file 6 — Additional file 6: Table S6. 4T1 timecourse flow data. [file 40425_2019_794_MOESM6_ESM.docx]

**Supplementary Table 6**

| **4T1** | **Day 14 (n=10)** | | **Day 18 (n=9)** | |
| --- | --- | --- | --- | --- |
| **T-cell Panel** | **mean** | **SE** | **mean** | **SE** |
| Live (%singlets) | 58.690 | 3.455 | 40.440 | 2.736 |
| CD45+ (%live) | 31.090 | 2.749 | 40.810 | 2.587 |
| CD3+ (%CD45+) | 7.496 | 0.939 | 7.358 | 0.326 |
| CD4+ (%CD45+) | 4.197 | 0.504 | 3.868 | 0.234 |
| Treg (%CD45+) | 0.850 | 0.158 | 0.676 | 0.019 |
| CD8+ (%CD45+) | 0.799 | 0.161 | 1.615 | 0.197 |
| NK (%CD45+) | 1.165 | 0.457 | 1.024 | 0.260 |
| **Myeloid Panel** | **mean** | **SE** | **mean** | **SE** |
| Live (%singlets) | 47.58 | 15.12 | 56.03 | 20.59 |
| CD45+(%live) | 26.34 | 7.76 | 31.21 | 12.06 |
| CD11b+ (%CD45+) | 75.84 | 3.53 | 75.20 | 1.87 |
| M-MDSC (%CD45) | 6.52 | 1.58 | 6.03 | 1.95 |
| Ly6G-Ly6Clo (%CD45) | 45.06 | 5.22 | 43.93 | 4.65 |
| Macrophages (%CD45+) | 36.55 | 5.02 | 36.02 | 5.85 |
| M1 like (%CD45+) | 6.06 | 1.63 | 3.95 | 0.93 |
| M2 like (%CD45+) | 7.66 | 2.05 | 6.39 | 2.21 |
| MHCII+CD206+ (%CD45) | 16.73 | 3.59 | 21.52 | 5.84 |
| MHCII-CD206- (%CD45) | 6.10 | 0.90 | 4.16 | 0.71 |
| G-MDSC (%CD45+) | 18.05 | 4.04 | 19.52 | 4.25 |
| DC (%CD45+) | 8.58 | 2.23 | 8.95 | 3.40 |
| B cells (%CD45+) | 0.56 | 0.24 | 0.40 | 0.15 |
