# Supplementary material for: Longitudinal immune characterization of syngeneic tumor models to enable model selection for immune oncology drug discovery
Source: J Immunother Cancer. 2019 Nov 28;7:328. doi: 10.1186/s40425-019-0794-7 (PMC6883640; doi:10.1186/s40425-019-0794-7)
Supplement: Supplementary file 7 — Additional file 7: Table S7. CT-26 checkpoint treatment flow data. [file 40425_2019_794_MOESM7_ESM.docx]

**Supplementary Table 7**

| **CT-26** | **Isotype Control** | | **mPD-L1 + mCTLA-4** | |  |
| --- | --- | --- | --- | --- | --- |
| **T-cell panel** | **mean** | **SE** | **mean** | **SE** | **pValue** |
| Live (%singlets) | 90.18 | 1.82 | 65.67 | 5.36 | <.0001 |
| CD45+ (%live) | 25.19 | 1.33 | 38.96 | 3.10 | 0.0001 |
| CD3+ (%CD45+) | 12.10 | 1.51 | 23.06 | 2.73 | 0.0008 |
| CD4+ (%CD45+) | 4.19 | 0.50 | 8.64 | 1.58 | 0.0071 |
| Treg (%CD45) | 1.27 | 0.15 | 1.74 | 0.22 | 0.0800 |
| CD8+ (%CD45+) | 5.37 | 0.99 | 9.64 | 1.02 | 0.0052 |
| NK+ (%CD45+) | 12.53 | 1.68 | 17.00 | 1.92 | 0.0878 |
| **Myeloid panel** |  | |  | |  |
| Live (%singlets) | 85.76 | 2.27 | 67.24 | 3.85 | 0.0002 |
| CD45+ (%live) | 21.86 | 2.64 | 35.79 | 3.50 | 0.0029 |
| B cells (%CD45+) | 0.90 | 0.32 | 1.46 | 0.33 | 0.2397 |
| CD11b+ (%CD45+) | 44.49 | 3.69 | 27.45 | 3.82 | 0.0030 |
| G-MDSC (%CD45+) | 7.21 | 1.33 | 4.30 | 1.13 | 0.1085 |
| M-MDSC (%CD45+) | 5.74 | 0.64 | 6.00 | 0.91 | 0.8066 |
| Ly6G-Ly6Clo (%CD45+) | 29.70 | 2.57 | 15.62 | 2.01 | 0.0002 |
| DC (%CD45+) | 0.69 | 0.13 | 0.37 | 0.12 | 0.0739 |
| Macrophages (%CD45+) | 18.32 | 2.64 | 6.48 | 1.50 | 0.0006 |
| M1 like(%CD45+) | 8.91 | 2.08 | 3.56 | 1.20 | 0.0377 |
| M2 like (%CD45+) | 2.39 | 0.65 | 0.31 | 0.11 | 0.0053 |
| MHC II+CD206+(%CD45+) | 5.25 | 0.64 | 1.15 | 0.24 | <.0001 |
| MHC II- CD206-(%CD45+) | 1.78 | 0.46 | 1.47 | 0.35 | 0.6023 |
