# Supplementary material for: Longitudinal immune characterization of syngeneic tumor models to enable model selection for immune oncology drug discovery
Source: J Immunother Cancer. 2019 Nov 28;7:328. doi: 10.1186/s40425-019-0794-7 (PMC6883640; doi:10.1186/s40425-019-0794-7)
Supplement: Supplementary file 8 — Additional file 8: Table S8. MC38 checkpoint treatment flow data. [file 40425_2019_794_MOESM8_ESM.docx]

**Supplementary Table 8**

| **MC38** | **Isotype Control** | | **mPD-L1 + mCTLA-4** | |  |
| --- | --- | --- | --- | --- | --- |
| **T-cell Panel** | **mean** | **SE** | **mean** | **SE** | **pValue** |
| Live (%singlets) | 90.89 | 1.42 | 83.70 | 2.87 | 0.0264 |
| CD45+(%live) | 49.26 | 1.88 | 65.22 | 1.60 | <.0001 |
| CD3+ (CD45+) | 6.08 | 0.42 | 9.14 | 0.65 | 0.0003 |
| CD4+ (%CD45+) | 2.21 | 0.09 | 3.39 | 0.28 | 0.0002 |
| Treg (CD45+) | 0.84 | 0.11 | 0.77 | 0.11 | 0.685 |
| CD8+ (%CD45+) | 1.31 | 0.17 | 3.28 | 0.43 | <.0001 |
| NK (%CD45+) | 5.64 | 0.55 | 6.84 | 0.98 | 0.2831 |
| **Myeloid Panel** | **Isotype Control** | | **mPD-L1 + mCTLA-4** | |  |
| Live (%singlets) | 92.61 | 1.11 | 86.51 | 2.32 | 0.0194 |
| CD45+ (%live) | 55.73 | 1.67 | 70.06 | 1.72 | <.0001 |
| B cells (%CD45+) | 0.34 | 0.04 | 0.39 | 0.08 | 0.5863 |
| CD11b+ (%CD45+) | 85.32 | 0.64 | 79.66 | 1.29 | 0.0003 |
| G-MDSC (%CD45+) | 0.67 | 0.09 | 0.95 | 0.14 | 0.0955 |
| M-MDSC (%CD45+) | 22.43 | 0.94 | 16.67 | 1.01 | 0.0002 |
| Ly6G-Ly6Clo (%CD45+) | 61.36 | 1.13 | 61.06 | 1.71 | 0.8806 |
| DC (%CD45+) | 2.58 | 0.49 | 2.57 | 0.50 | 0.9826 |
| Macrophages (%CD45+) | 50.41 | 1.12 | 47.99 | 2.35 | 0.3438 |
| M1 like(%CD45+) | 16.45 | 2.04 | 15.77 | 1.96 | 0.8132 |
| M2 like (%CD45+) | 7.16 | 1.60 | 8.08 | 1.79 | 0.7014 |
| MHC II+CD206+(%CD45+) | 7.87 | 1.79 | 14.38 | 3.06 | 0.0682 |
| MHC II- CD206-(%CD45+) | 18.91 | 2.86 | 9.75 | 1.14 | 0.007 |
