# Supplementary material for: Longitudinal immune characterization of syngeneic tumor models to enable model selection for immune oncology drug discovery
Source: J Immunother Cancer. 2019 Nov 28;7:328. doi: 10.1186/s40425-019-0794-7 (PMC6883640; doi:10.1186/s40425-019-0794-7)
Supplement: Supplementary file 9 — Additional file 9: Figure S1. Impact of α-mPD-L1+ α -mCTLA-4 treatment on survival in syngeneic models. [file 40425_2019_794_MOESM9_ESM.pptx]

## Slide 1
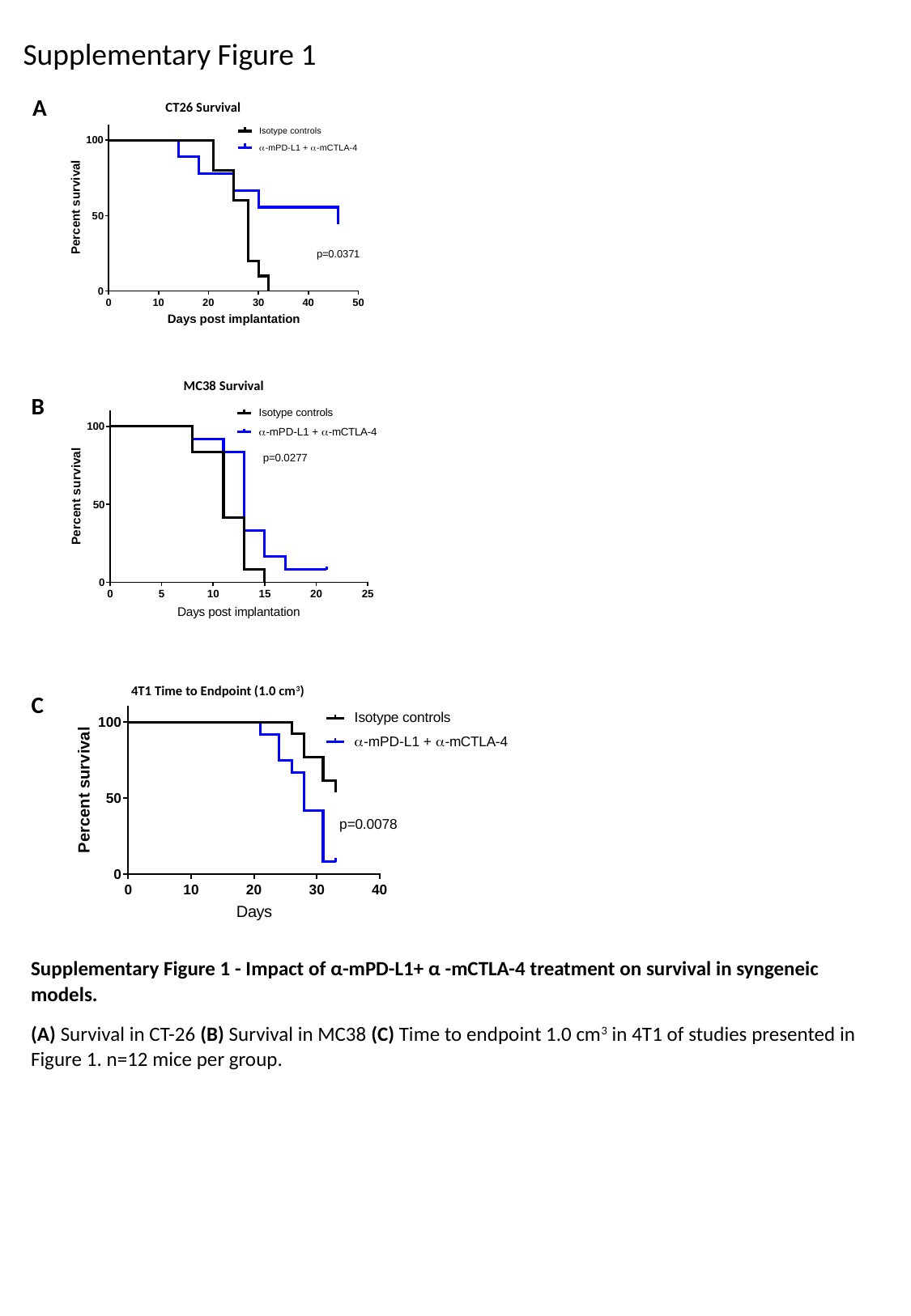

Supplementary Figure 1
A
CT26 Survival
MC38 Survival
B
4T1 Time to Endpoint (1.0 cm3)
C
Supplementary Figure 1 - Impact of α-mPD-L1+ α -mCTLA-4 treatment on survival in syngeneic models.
(A) Survival in CT-26 (B) Survival in MC38 (C) Time to endpoint 1.0 cm3 in 4T1 of studies presented in Figure 1. n=12 mice per group.
