# Supplementary material for: Longitudinal immune characterization of syngeneic tumor models to enable model selection for immune oncology drug discovery
Source: J Immunother Cancer. 2019 Nov 28;7:328. doi: 10.1186/s40425-019-0794-7 (PMC6883640; doi:10.1186/s40425-019-0794-7)
Supplement: Supplementary file 10 — Additional file10: Figure S2. Expression of PD-L1 on CD11b+ and CD45- cells. [file 40425_2019_794_MOESM10_ESM.pptx]

## Slide 1
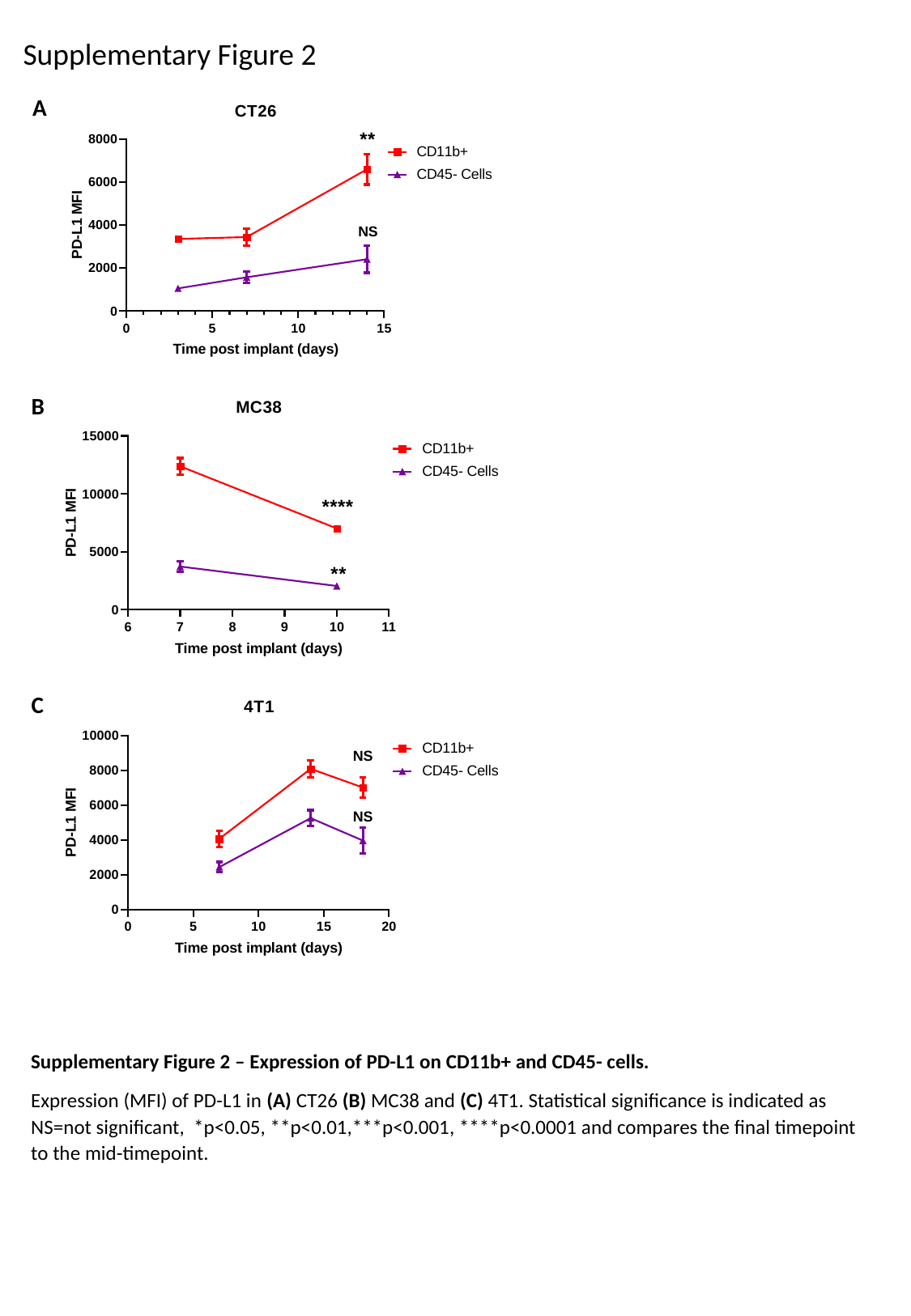

Supplementary Figure 2
A
B
C
Supplementary Figure 2 – Expression of PD-L1 on CD11b+ and CD45- cells.
Expression (MFI) of PD-L1 in (A) CT26 (B) MC38 and (C) 4T1. Statistical significance is indicated as NS=not significant, *p<0.05, **p<0.01,***p<0.001, ****p<0.0001 and compares the final timepoint to the mid-timepoint.
