# Supplementary material for: Longitudinal immune characterization of syngeneic tumor models to enable model selection for immune oncology drug discovery
Source: J Immunother Cancer. 2019 Nov 28;7:328. doi: 10.1186/s40425-019-0794-7 (PMC6883640; doi:10.1186/s40425-019-0794-7)
Supplement: Supplementary file 11 — Additional file 11: Figure S3. Protein expression changes measured by NPX [file 40425_2019_794_MOESM11_ESM.pptx]

## Slide 1
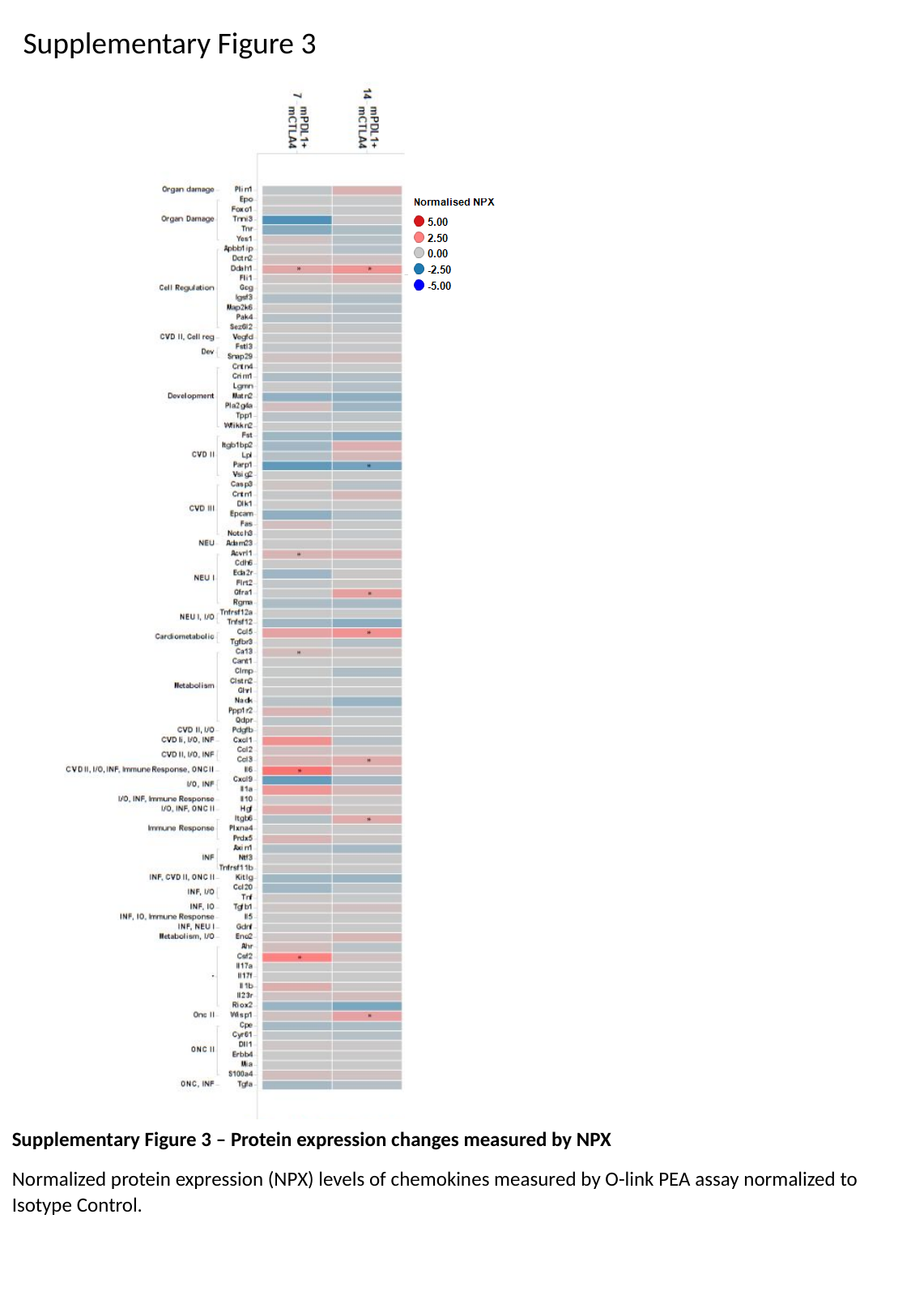

Supplementary Figure 3
Supplementary Figure 3 – Protein expression changes measured by NPX
Normalized protein expression (NPX) levels of chemokines measured by O-link PEA assay normalized to Isotype Control.
