# Supplementary material for: Longitudinal immune characterization of syngeneic tumor models to enable model selection for immune oncology drug discovery
Source: J Immunother Cancer. 2019 Nov 28;7:328. doi: 10.1186/s40425-019-0794-7 (PMC6883640; doi:10.1186/s40425-019-0794-7)
Supplement: Supplementary file 12 — Additional file 12: Figure S4. Impact of tumor size on gene expression changes in CT-26 [file 40425_2019_794_MOESM12_ESM.pptx]

## Slide 1
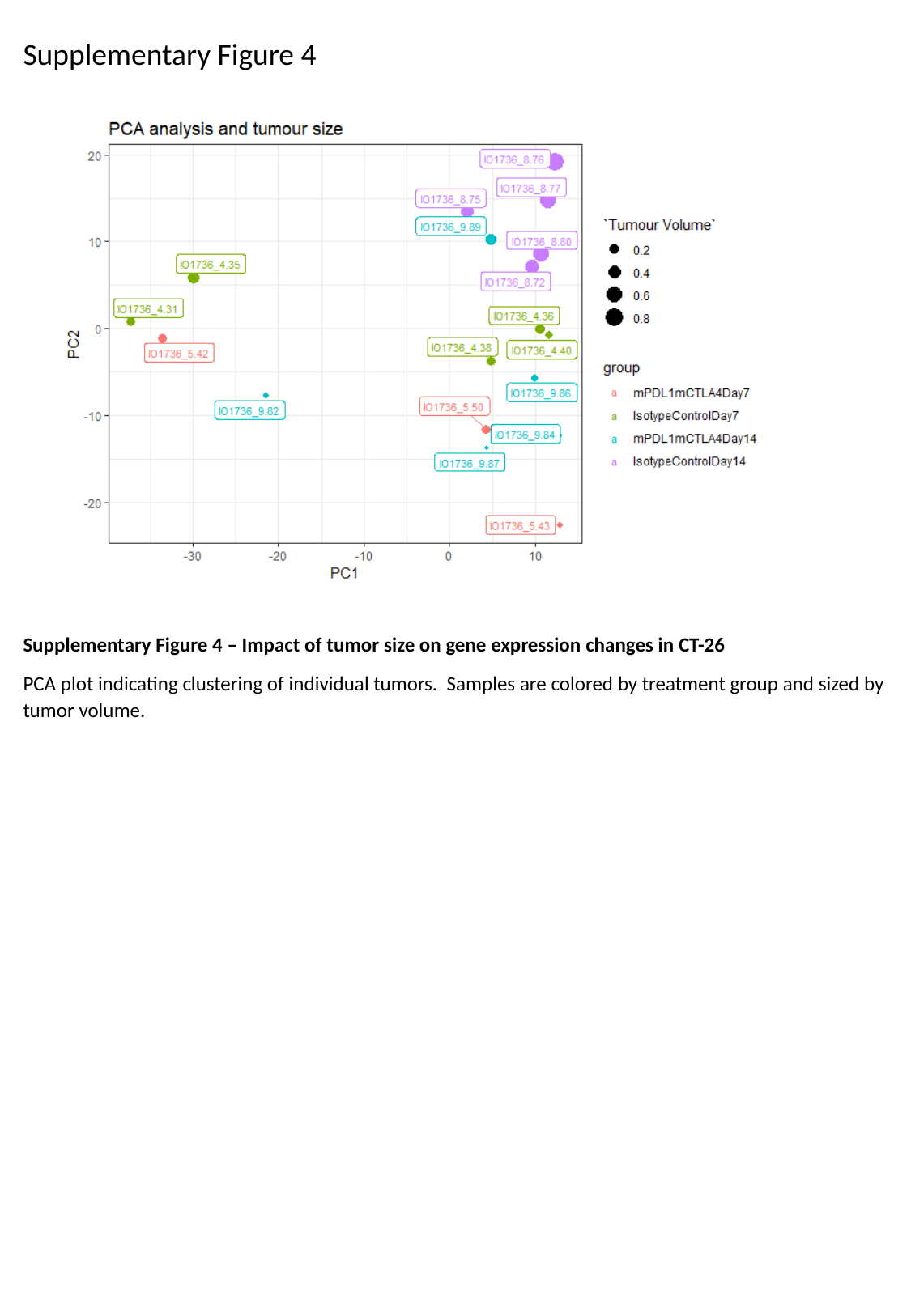

Supplementary Figure 4
Supplementary Figure 4 – Impact of tumor size on gene expression changes in CT-26
PCA plot indicating clustering of individual tumors. Samples are colored by treatment group and sized by tumor volume.
