# Supplementary material for: Longitudinal immune characterization of syngeneic tumor models to enable model selection for immune oncology drug discovery
Source: J Immunother Cancer. 2019 Nov 28;7:328. doi: 10.1186/s40425-019-0794-7 (PMC6883640; doi:10.1186/s40425-019-0794-7)
Supplement: Supplementary file 13 — Additional file 13: Figure S5. Immune cell content in CT-26 responders and non-responders [file 40425_2019_794_MOESM13_ESM.pptx]

## Slide 1
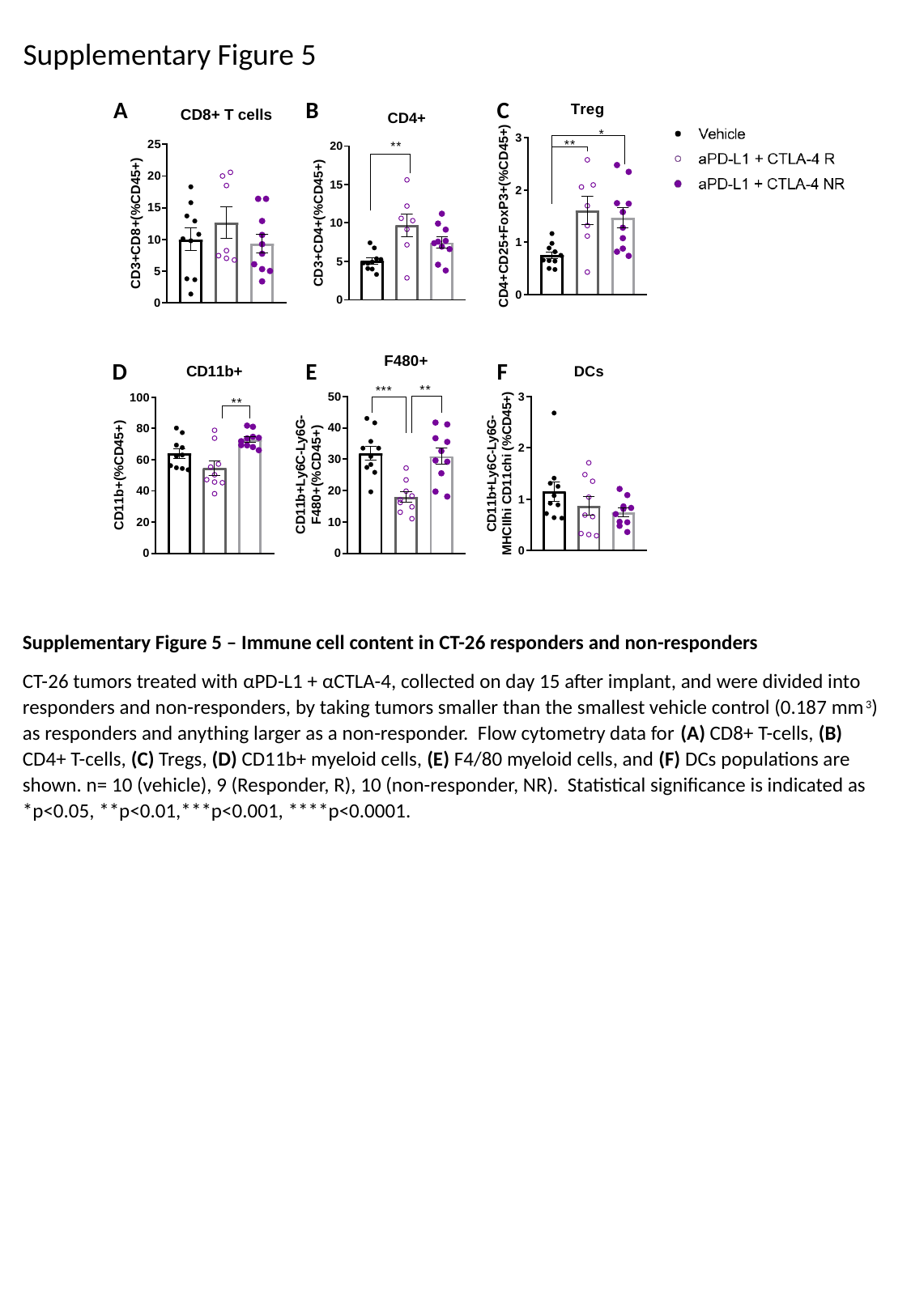

Supplementary Figure 5
A
B
C
D
E
F
Supplementary Figure 5 – Immune cell content in CT-26 responders and non-responders
CT-26 tumors treated with αPD-L1 + αCTLA-4, collected on day 15 after implant, and were divided into responders and non-responders, by taking tumors smaller than the smallest vehicle control (0.187 mm3) as responders and anything larger as a non-responder. Flow cytometry data for (A) CD8+ T-cells, (B) CD4+ T-cells, (C) Tregs, (D) CD11b+ myeloid cells, (E) F4/80 myeloid cells, and (F) DCs populations are shown. n= 10 (vehicle), 9 (Responder, R), 10 (non-responder, NR). Statistical significance is indicated as *p<0.05, **p<0.01,***p<0.001, ****p<0.0001.
